# Fuel layer specific pollutant emission factors for fire prone forest ecosystems of the western U.S. and Canada

**DOI:** 10.1016/j.aeaoa.2022.100188

**Published:** 2022-12

**Authors:** Shawn P. Urbanski, Russell W. Long, Hannah Halliday, Emily N. Lincoln, Andrew Habel, Matthew S. Landis

**Affiliations:** a U.S.D.A. Forest Service, Rocky Mountain Research Station, Missoula, MT, USA; b US EPA, Office of Research and Development, Research Triangle Park, NC, USA; c Jacobs Technology Inc, Research Triangle Park, NC, USA

**Keywords:** Emission factors, Wildland fire smoke, Prescribed fire, PM_2.5_, Methane, Carbon, Volatile organic compounds

## Abstract

Wildland fires are a major source of gases and aerosols, and the production, dispersion, and transformation of fire emissions have significant ambient air quality impacts and climate interactions. The increase in wildfire area burned and severity across the United States and Canada in recent decades has led to increased interest in expanding the use of prescribed fires as a forest management tool. While the primary goal of prescribed fire use is to limit the loss of life and property and ecosystem damage by constraining the growth and severity of future wildfires, a potential additional benefit of prescribed fire - reduction in the adverse impacts of smoke production and greenhouse gas (GHG) emissions - has recently gained the interest of land management agencies and policy makers in the United States and other nations. The evaluation of prescribed fire/wildfire scenarios and the potential mitigation of adverse impacts on air quality and GHGs requires fuel layer specific pollutant emission factors (EFs) for fire prone forest ecosystems. Our study addresses this need with laboratory experiments measuring EFs for carbon dioxide (CO_2_), carbon monoxide (CO), methane (CH_4_), ethyne (C_2_H_2_), formaldehyde (H_2_CO), formic acid (CH_2_O_2_), hydrogen cyanide (HCN), fine particulate matter (PM_2.5_), nitric oxide (NO), nitrogen dioxide (NO_2_), sulfur dioxide (SO_2_), and total reduced sulfur (TRS) for the burning of individual fuel components from three forest ecosystems which account for a large share of wildfire burned area and emissions in the western United States and Canada - Douglas fir, ponderosa pine, and black spruce/jack pine.

## 1. Introduction

Wildland fire (wildfires and prescribed fires) smoke contains hundreds of gases (Urbanski 2014; Hatch et al., 2015) and aerosols diverse in size, composition, and morphology (Reid et al., 2005a, b). Globally and in the United States (U.S.), wildland fires are a major source of gases and aerosols (Bond et al., 2013; van der Werf et al., 2017), and the production, dispersion, and transformation of fire emissions have significant air quality impacts and climate interactions. Wildfire smoke can trigger severe, multi-week pollution episodes over large areas with substantial impacts on public health (Reisen et al., 2015; Cascio, 2018). Wildland fires are a major source of fine particulate matter (PM_2.5_; particulates with an aerodynamic diameter <2.5 μm) (Lu et al., 2016; Brey et al., 2018) and can contribute to downwind secondary ozone (O_3_) production (McClure and Jaffe, 2018), both of which are criteria ambient air pollutants regulated under the U.S. Clean Air Act. In addition to carbon dioxide (CO_2_), wildland fires produce large amounts of methane (CH_4_), an important greenhouse gas (GHG). CH_4_ has a 100-y global warming potential (GWP) that is ~32 times that of CO_2_, and it is a major contributor to increases in tropospheric O_3_, itself a GHG (Nisbet et al., 2020).

Understanding the composition and magnitude of smoke emissions is vital for addressing the range of decision support needs initiated by wildland fire smoke. Accurately characterizing the dependence of emissions on fuels, fire behavior, and environmental conditions is key to improving basic smoke management practices and facilitating the use of prescribed fire. Emissions are essential input to smoke forecasting systems relied upon by public health officials, air quality forecasters, and fire management teams to mitigate the impacts of wildland fire smoke on public health and safety. Emission factors (EFs) quantify the relative abundance of pollutants in fresh smoke and are an essential input to national emission calculations that drive a range of smoke forecasting systems (Larkin et al., 2009; Larkin, 2018; NOAA, 2021) and emission inventories (U.S. EPA, 2021). The smoke management tools employed by land managers for planning prescribed burns, such as the First Order Fire Effects Model (FOFEM; Lutes, 2019), also require EFs for emissions calculations. EFs depend on many factors including fire behavior and fuel properties such as the structure and arrangement of fuels (e.g., size, shape, and packing of fuel particles, fuel condition) moisture content and growth stage (Peterson et al., 2021). Within a given ecosystem, EFs can differ substantially across the various fuel components that may be present such as tree canopy, grass, shrubs, litter, fine dead wood, logs, and duff/peat (Peterson et al., 2021). The extent to which different fuel components are consumed by fire is highly variable. A low intensity broadcast prescribed fire may consume only litter, fine dead wood, and grasses (Agee and Skinner, 2005) while a high intensity wildfire may torch tree canopies across 1000s of hectares (ha) and consume the surface fuels and forest floor down to the mineral soil (Morgan et al., 2014).

The increase in the number of large wildfires (>500 ha) and area burned over recent decades (Westerling 2016; Holden et al., 2018), the increased likelihood of large, high severity wildfires in the future due to a history of fire suppression, increased frequency and severity of drought, warmer temperatures (Kitzberger et al., 2007; Littell et al., 2009; U.S. Department of United States Department of Agriculture, 2014; Abatzoglou and Williams 2016; Westerling et al., 2016), and growth of the wildland-urban interface has led to increased interest in expanding the use of prescribed fires as a forest management tool (Ager et al., 2104; Stephens et al., 2021). Prescribed fire mitigates wildfire hazard through the reduction of hazardous fuels and by restoring and maintaining ecosystem health (Agee and Skinner, 2005). Despite the potential benefits, the expanded use of prescribed fire faces many barriers. Smoke management concerns (e.g., visibility, nuisance) are among the top impediments to prescribed burning (Melvin, 2015, 2018). Reducing smoke concerns as an obstacle to expanded prescribed fire use depends in part on improved smoke predictions tools. With respect to prescribed fire in western forests of the U.S. and Canada, improving smoke prediction tools requires better characterized EFs for prescribed fires.

Recently, significant progress has been achieved in quantifying EFs for wildfires in western ecosystems (Koss et al., 2018; Selimovic et al., 2018; Permar et al., 2021). However, these recent studies have focused on wildfires and provide EFs which may not be directly applicable to western prescribed fires. Broadcast prescribed burns in forests are usually planned to remove light surface fuels (litter and fine woody debris) and understory fuels (shrubs and grasses) that spread surface fire and initiate crown fire. Prescribed fire objectives in forests most often avoid canopy fire and sustained burning of duff/peat since this fire behavior results in tree mortality. In contrast, fire average EFs measured for western wildfires (measured in the field or laboratory simulated fires) include emissions from fuel strata not commonly burned in prescribed fires (canopy, duff).

In addition to improving smoke modeling tools, prescribed fire specific EFs may also improve our (researchers, land managers, and policy makers) understanding of prescribed fire impacts on future emissions. While the primary goal of prescribed fire use is to limit the loss of life and property and ecosystem damage by constraining the growth and severity of future wildfires (Agee and Skinner, 2005), a potential additional benefit that has recently gained the interest of land management agencies and policy makers is the reduction in the adverse public health impacts of wildfire smoke production and GHG emissions (Loudermilk et al., 2014; Volkova et al., 2014; Williamson et al., 2016). Characterizing the net impacts of prescribed fire on air quality and GHG emissions (i.e., present day emissions from prescribed fire *versus* avoided future wildfire emissions) requires emission estimates for the individual fuel components burned differentially in prescribed fire and wildfires. EFs for specific fuel layers are needed to estimate emissions for the evaluation of prescribed fire/wildfire scenarios and the potential mitigation of adverse impacts on ambient air quality and GHGs. Our study addresses this need by reporting results from laboratory experiments measuring pollutant EFs for the burning of individual fuel components from Douglas fir, ponderosa pine, and black spruce/jack pine forest ecosystems which account for a large share of wildfire burned area and emissions in the western U.S. and Canada. This study provides EFs for litter and fine woody debris (components targeted by prescribed fire) and canopy fuels (generally not burned in prescribed fires) that may serve as a starting point for quantifying emission trade-offs associated with prescribed fire use.

## 2. Experimental

### 2.1. USDA Forest Service Fire Sciences Laboratory

**Fig. 1.**
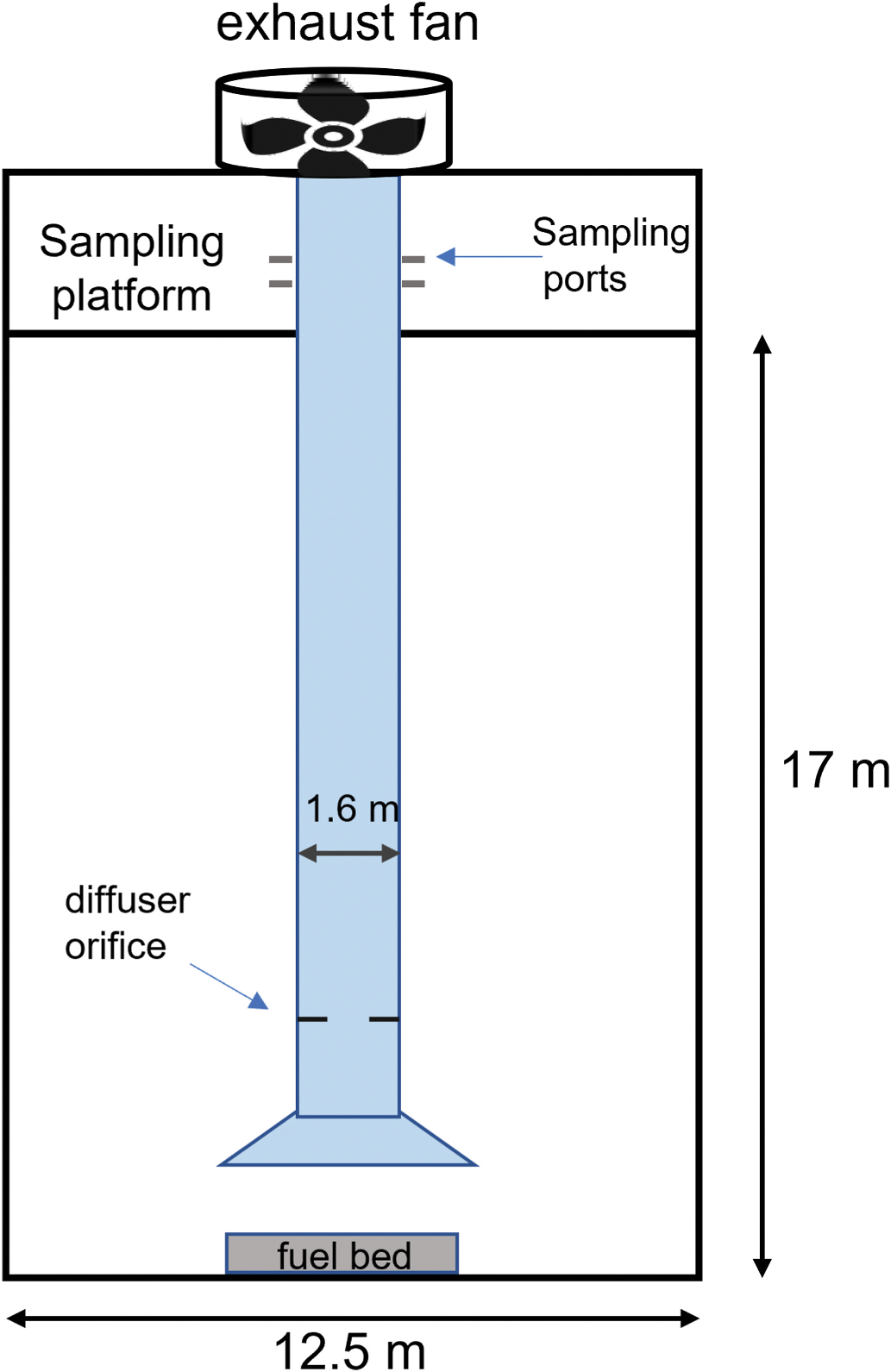
Schematic of USDA Forest Service combustion chamber.

The study was conducted in the large-scale combustion chamber at the U.S. Department of Agriculture (USDA) Forest Service Fire Sciences Laboratory (FSL), which is depicted in Fig. 1. The 3280 m^3^ combustion chamber (dimensions 12.5 m × 12.5 m × 21 m) has an exhaust stack (d = 1.6 m) with an inverted funnel (d = 3.5 m) that extends from 2 m above the floor to the top of the chamber. A sampling platform surrounds the stack 17 m above the chamber floor. At the level of the platform the exhaust stack has customizable access ports and hatches for sampling emissions (gas and aerosol) and monitoring flow conditions. Multiple researchers and instruments can be deployed on the sampling platform (2200-kg capacity). During emissions experiments, outdoor air from vents at the base of the chamber walls is drawn through the stack and entrains emissions from fires burning in fuelbeds assembled directly beneath the funnel. Within the exhaust stack is a diffuser ring (i.d. = 0.8 m) that mixes the air and entrained emissions to provide uniform temperature and mixing ratio across the width of the stack at the height of the sampling platform (Christian et al., 2003, 2004). A viewing room connected to the combustion chamber allows multiple researchers to observe and document experiments without perturbing conditions within the chamber.

In our experiments the gas and particle measurement instruments were positioned on the platform and sampled emissions drawn through sample lines (see Section 2.3 for details). Fuelbeds were assembled directly under the exhaust stack on a high-temperature ceramic fiber board. Fuels were ignited using a propane lighter. Some of the high moisture content litter burns required a small amount of ethanol (1–2 g) on the fuelbed edge to achieve ignition. Burn durations ranged from 3.5 to 37 min varying with the type, amount, and condition (moisture content) of fuel. The end of a burn was called when the carbon monoxide (CO) mixing ratio decreased below 0.5 ppm.

### 2.2. Fuels

Montane fuels were harvested in April 2021 in four geographic areas around Missoula, Montana on the Lolo National Forest. Three types of coniferous species were selected: ponderosa pine (Pinus ponderosa) and Douglas fir (Pseudotsuga menziesii), and western larch (Larix occidentalis). Multiple fuel types of each species were collected including downed needles, fine woody debris (FWD; dead branches, twigs <5 cm diameter), cones, litter (undecomposed or only partially decomposed organic material) and canopy fuels - green branches (boughs with live needles and branches <3 cm). Douglas fir green branches and moist ponderosa pine needles were collected along the Point Six road (47°0′17.59”N, 114° 1′21.14”W). Western larch needles, FWD, and litter and ponderosa pine needles were collected in the Ninemile area (47°6′43.51”N, 114°24′6.44”W). Douglas fir litter and FWD was collected in the Albert creek area. (46°58′12.83”N, 114°14′39.58”W). Ponderosa pine needles, FWD and cones were collected on the O’Brien creek area (46°51′1.46”N, 114°11′21.89”W).

The black spruce (*Picea mariana*) and jack pine (*Pinus banksiana*) fuels were harvested by personnel from the Wood Buffalo Environmental Association (WBEA) in October 2019 near their forest health monitoring program jack pine site 2054 (57°6′55.73”N, −111°25′48.25”W), black spruce site 2554 (57°6′58.98”N, −111°25′42.36”W), and at an atmospheric deposition black spruce site 4000 (56°19′45.98”N, −111°35′19.03”W) in Alberta, Canada (Foster et al., 2019). Dead branches with needles were cut from the top side of recently fallen trees. WBEA sites 2054 and 2554 are located ~15 km southeast of Fort McKay in an area shown to be impacted by enhanced total sulfur and total nitrogen deposition and site 4000 is located ~42 km southwest of Fort McMurray in an area of lower atmospheric deposition (Edgerton et al., 2020). Enhanced atmospheric deposition in the region has been demonstrated to correlate with total nitrogen and total sulfur foliar concentrations (MacKenzie and Dietrich, 2020).

Ponderosa pine surface fuelbeds were constructed using a fuel amount and area that provided loadings (mass/area) similar to western ponderosa pine forests (O’Connell et al., 2016; Urbanski et al., 2018). We used a fuelbed area of 30 cm × 46 cm. With the fuelbed area fixed, burns with larger total fuel mass had deeper fuelbeds allowing us to mimic the natural variability of litter and FWD fuel loading. The freshly harvested, green canopy fuels were burned by placing the boughs over a small bed of dry ponderosa pine needles (20–50 g) which were ignited with a propane lighter. Canopy fuelbeds were arranged using two configurations: for the first several burns, boughs were laid directly on top of the needles while for the remaining burns boughs were suspended several centimeters above the needle bed. The canopy fuelbed setup was chosen to mimic the initiation of torching and active crown fire in wildfires where fire in the surface fuels ignites the canopy (http://www.firewords.net/definitions/crown_fire.htm). The moisture content of fuels, reported as a percent of dry mass, was determined by oven drying fuel samples at 70 °C for 48 h. Fuels information for all burns is provided in Appendix Table A.1. Fuel samples were sent to Cumberland Valley Analytical Services (Waynesboro, PA, USA) for determination of total carbon, nitrogen, and sulfur content. Analytical results are summarized by fuel type in Appendix Table A.2.

Our study focused on five fuelbeds - three variations of ponderosa pine surface fuels, Douglas fir canopy, and black spruce/jack pine surface fuels. The ponderosa pine fuelbeds were needles only, needles and FWD, and needles and cones and are referred to as PPN, PPN + PPW, and PPN + PPC, respectively. The Douglas fir canopy and black spruce/jack pine fuelbeds are referred to as “canopy” and “Alberta”. These fuelbeds were selected to address gaps in the current emissions literature. We note that emissions from ponderosa pine and black spruce canopy fuels have been previously reported in Stockwell et al. (2014) and we therefore have limited our canopy fuel focus to Douglas fir.

### 2.3. Instrument details

The instruments utilized during the emissions experiments are listed in Table 1. All instruments, except the LI-COR, were positioned on the stack sampling platform (Fig. 1) and sampled smoke from the exhaust stack through lines constructed of stainless steel or perfluoroalkoxy (PFA) Teflon^™^. The LI-COR was positioned on the combustion chamber floor at the edge of the inverted funnel (Fig. 1) to monitor background CO_2_ concentrations during burns. All Teledyne API, Thermo Scientific, and LI-COR continuous gas analyzers were zeroed, and span calibrated at the beginning and end of each chamber test day using certified Teledyne API Model T700U dynamic dilution calibration systems. EPA protocol certified gas standard cylinders diluted in ultra-scientific grade zero air were used for NO, NO_2_, NO_x_, SO_2_ and TRS instruments. Multi-point span calibrations were conducted every two days of testing to ensure linearity.

The CRDS gas analyzer provided high time resolution (2 s) concentration measurements. Details of the CRDS analyzer and its implementation for measuring of biomass burning emissions have been previously described by Urbanski (2013). The CRDS analyzer response was stable over the 11 days of experiments as confirmed with a three-point calibration using gas mixtures of CO_2_, CO, and CH_4_ in scientific grade zero air conducted prior to and after the experiments.

**Appendix Fig. B.1.**
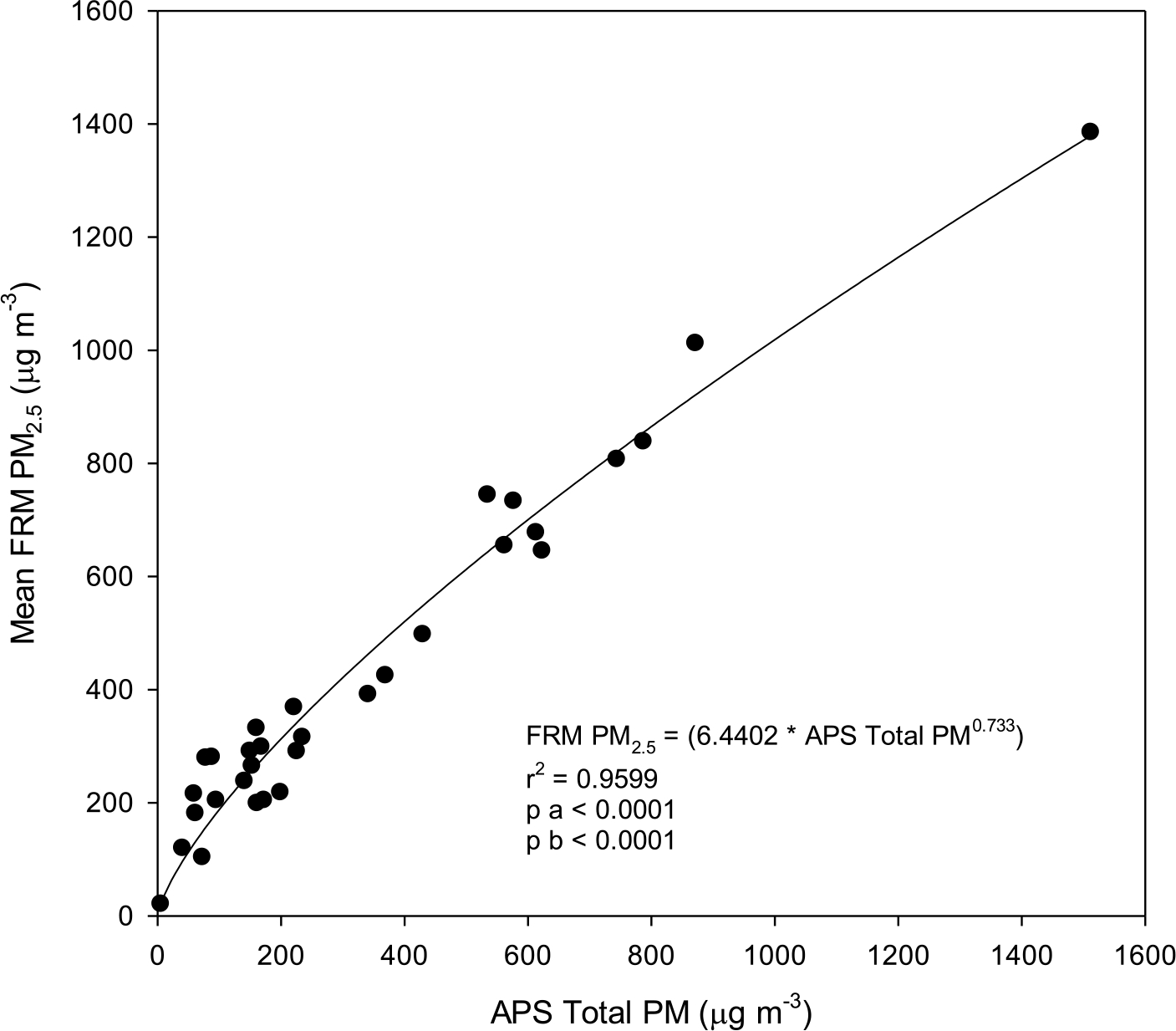
APS Total PM versus FRM PM_2.5_ Power Model Calibration Equation.

The TSI (Shoreview, MN, USA) Model 3321 APS provided 10 s resolution particle differential number concentration (dN) and differential mass concentration (dM) in 52 size channels ranging from 0.5 to 20 μm (Peters et al., 2006; Peters, 2006). Converting aerosol number concentration to particle mass concentrations requires assumptions for aerodynamic shape factor and density (Peters, 2006) for which we could find no smoke specific information in the literature. As a result, a power function calibration model was developed and utilized between the APS total particulate matter concentration and the Tisch Environmental (Cleves, OH, USA) model TE-WILBUR filter-based 1 h Federal Reference Sampler (FRM) PM_2.5_ concentration in a manner similar to that described by Landis et al. (2021) during static burn testing completed prior to stack testing (see Appendix B; Appendix Figure B1).

The Tunable Infrared Laser Direct Absorption Spectroscopy (TILDAS) instrument employed dual quantum cascade tunable lasers in the mid infrared (IR) range (Herndon et al., 2007; McManus et al., 2015) to measuring formaldehyde (H_2_CO) and formic acid (CH_2_O_2_) at 1764–1766 cm^−1^, and acetylene (C_2_H_2_) and hydrogen cyanide (HCN) at 3286–3290 cm^−1^. The spectrometer measures light attenuation over a 76 m astigmatic multi-pass cell operated at 50 torr. Collected spectra were averaged to 1 s and fit with non-linear least squares algorithms based on the high-resolution transmissions HITRAN database. The absorption measurements were made relative to zero air introduced to the inlet from a Teledyne API model T701 zero air generator. Automated zero reference measurements were conducted every 5 m for 10 s (with a 15 s flush times). All inlet materials where PFA, and the inlet used two sequential Millipore (Burlington, MA, USA) polytetrafluoroethylene (PTFE) membrane filters (5 μm and 1 μm pressure drop equivalents) to protect the instrument optics from smoke particles generated in the burn chamber. All standards and zeros were sampled through the 1 μm Teflon filter. Standards were introduced once per sampling day to track instrument performance and assess the active fitting parameters. A multicomponent mix of C_2_H_2_, H_2_CO, and HCN at 1 ppm (Apel-Reimer Environmental, Inc., Miami, FL, USA) was dynamically diluted with zero air to perform multipoint calibration checks, and two permeation tubes continuously purged with nitrogen were used to further assess the stability of H_2_CO and CH_2_O_2_. All calibration materials where continuously flushed to keep all flow control equipment (regulators, mass flow controllers) equilibrated. Total uncertainty of all measurements is reported at 15% for the 1 s measurements.

### 2.4. Statistical analysis

Data integration, processing, and all statistical analyses were performed using SAS v.9.4 (SAS Institute, Cary, NC, USA) and R Statistical Software (v4.1.2; R Core Team 2021). The assumptions of the parametric procedures were examined using residual plots, skewness and kurtosis coefficients, Shapiro-Wilk test, and the Brown-Forsythe test. A level of significance of α = 0.05 was used for all statistical procedures unless otherwise stated. The R function *pairwise.wilcox.test* (using the Bonferroni p-value correction) from the base package stats was used for non-parametric paired EF comparison tests (hereafter Wilcoxon test (Hogg and Tanis, 2006)); after it was determined that the test data violated parametric test assumptions. TILDAS (1 s), NDIR (1 s), and CRDS (2 s) data files were logged using dedicated computer systems, and the Teledyne API and ThermoScientific instrument data were logged using an Envidas (Granville, OH, USA) Ultimate data acquisition system at 10 s resolution. All summary statistics and emission factors are based on the final integrated 10 s data set.

### 2.5. Emission calculations

The basic metric used to quantify fire emissions is the excess mixing ratio, which for species X is defined as $\Delta \text{X}={\text{X}}_{\text{smoke}}-{\text{X}}_{\text{background}}$, where ${\text{X}}_{\text{smoke}}$ and ${\text{X}}_{\text{background}}$ are the mixing ratio of X in fresh smoke and background air, respectively. For each burn, we calculated the average ΔX of each species from the 10 s data. The gas phase instruments reported volume mixing ratios which were converted to mass mixing ratio at standard conditions of temperature (298 K) and pressure (1 atm). Burn average EFs for each compound X, EFX (in units g of X per kg of dry fuel burned), were calculated from the burn average ΔX using the carbon mass balance method (Ward and Radke, 1993) implemented with Equation (1). In Equation (1), ΔC_T_ is the sum of the excess mass mixing ratios of carbon in each species, $MM_{X}$ is the molar mass of *X* (g mole^−1^), 12 is the molar mass of carbon (g mole^−1^), and $F_{c}$ is the mass fraction of carbon in the dry biomass (see Appendix Table A.2). We assumed the carbon mass fraction of PM_2.5_ was 0.67 (Burling et al., 2011). The carbon mass balance method assumes all biomass carbon that is volatilized as gases and aerosol is measured as excess mass mixing ratios and included in the denominator sum of Equation (1). While our study measured carbon in only seven gases and PM_2.5_, this results in only a minor overestimate of EFs as most of the carbon (>95%) in biomass smoke is contained in CO_2_, CO, and CH_4_ (Urbanski, 2014). Additional assumptions of the carbon mass balance method are uniform mixing of all smoke components and constant background composition.

(1) $$EFX=F_{c}\times 1000\left(gkg^{-1}\right)\times \frac{MM_{X}}{12}\times \frac{\Delta X}{\Delta C_{T}}$$

where: $\Delta C_{T}=\Delta CO_{2}+\Delta CO+\Delta CH_{4}+\Delta H_{2}CO+\Delta CH_{2}O_{2}+\Delta C_{2}H_{2}+\Delta HCN+\Delta PM_{2.5}$

The chemical composition of emissions from wildland fires are related to the combustion characteristics of the fire, in particular the relative amounts of flaming and smoldering combustion (Urbanski, 2014). Modified combustion efficiency (MCE; Equation (2)), the fraction of volatilized fuel carbon emitted as CO_2_ versus CO_2_ + CO, is used to characterize the relative amount of flaming and smoldering combustion (Ward and Radke, 1993; Akagi et al., 2011). MCE approaches 0.99 for pure flaming combustion e.g., fine fuels completely engulfed in flame (Chen et al., 2007; Yokelson et al., 1997), while MCE ~ 0.80 is typical for pure smoldering (Akagi et al., 2011). Since many species are predominantly associated with either the flaming or smoldering phases of combustion, the EF of many compounds correlate with MCE. Linear regressions of EF versus MCE have been useful for extrapolating laboratory measured EF to real-fire conditions (e.g., Selimovic et al., 2018). Given the utility of MCE for characterizing combustion characteristics and its potential for estimation of EF, we have calculated average MCE for all burns.


(2) $$MCE=\frac{\Delta CO_{2}}{\Delta CO_{2}+\Delta CO}$$

## 3. Results and discussion

### 3.1. Summary of EF

A total of 55 burns were conducted using ponderosa pine (n = 28) and black spruce/jack pine (Alberta fuels) (n = 11) surface fuels, and Douglas fir canopy (n = 16). One Douglas fir canopy burn was not used in our analysis because sustained burning did not occur, and fuel consumption was negligible. Three burns using mixtures of Larch and Douglas fir litter were also conducted, but due to the small sample size these burns are not included in our analysis. Three assortments of ponderosa pine fuels were used: needles only (n = 14), needles and fine woody debris (FWD) (n = 9), and needles and cones (n = 5). These ponderosa pine fuelbeds are hereafter referred to as PPN, PPN + PPW, and PPN + PPC, respectively. Excess mixing ratios of CO_2_, CO, CH_4_, C_2_H_2_, H_2_CO, CH_2_O_2_, HCN, NO, NO_2_, SO_2_, TRS, and PM_2.5_ measured over the duration of each burn were used to derive burn average EFs. Due to a temporary software issue we lacked TILDAS data for the first 13 burns. EFTRS are not reported for 8 burns due to insufficient signal-to-noise.

**Fig. 2.**
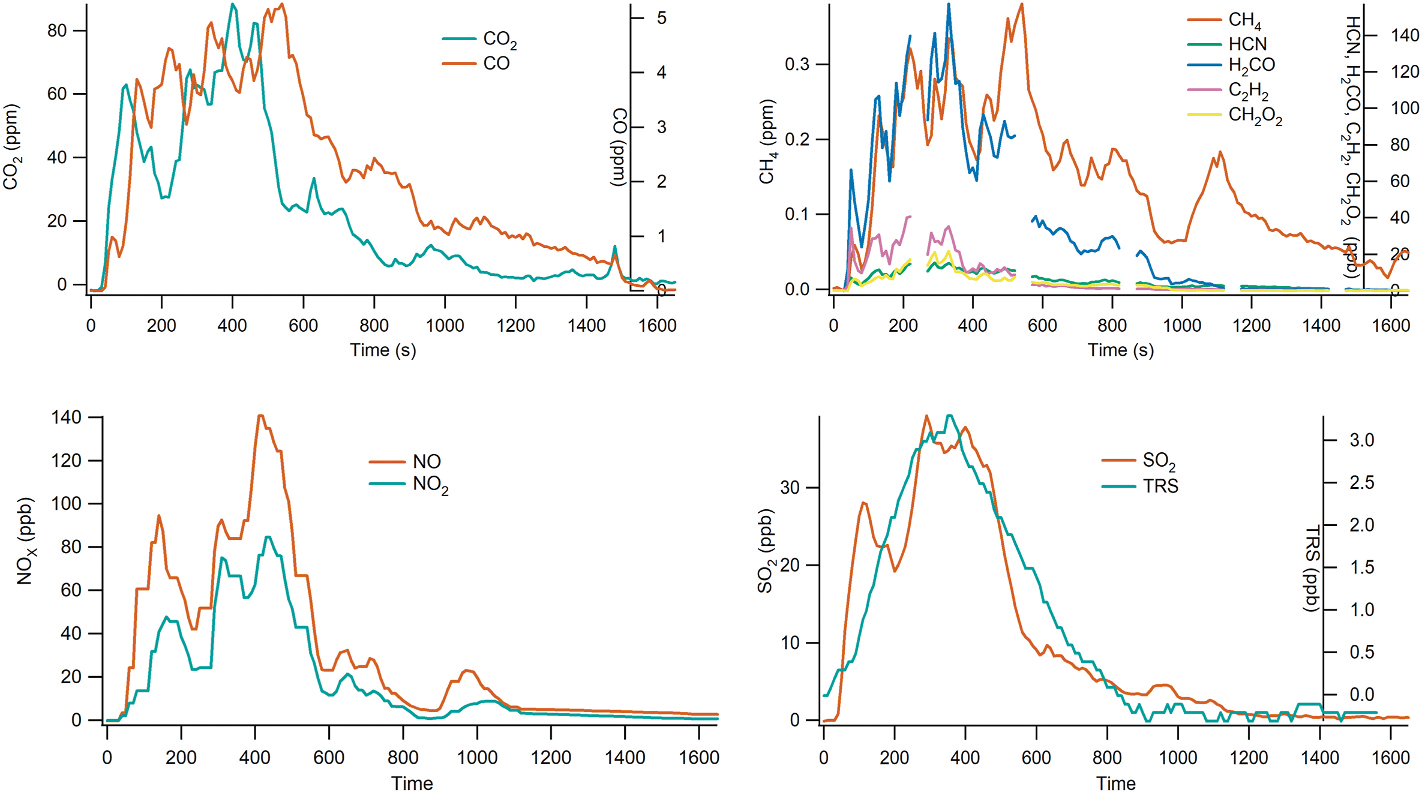
Excess mixing ratio time series for Burn 51, PPN + PPW. Dropouts in the TILDAS data (upper right panel) are due to automatic zero (see Section 2.2).

**Fig. 3.**
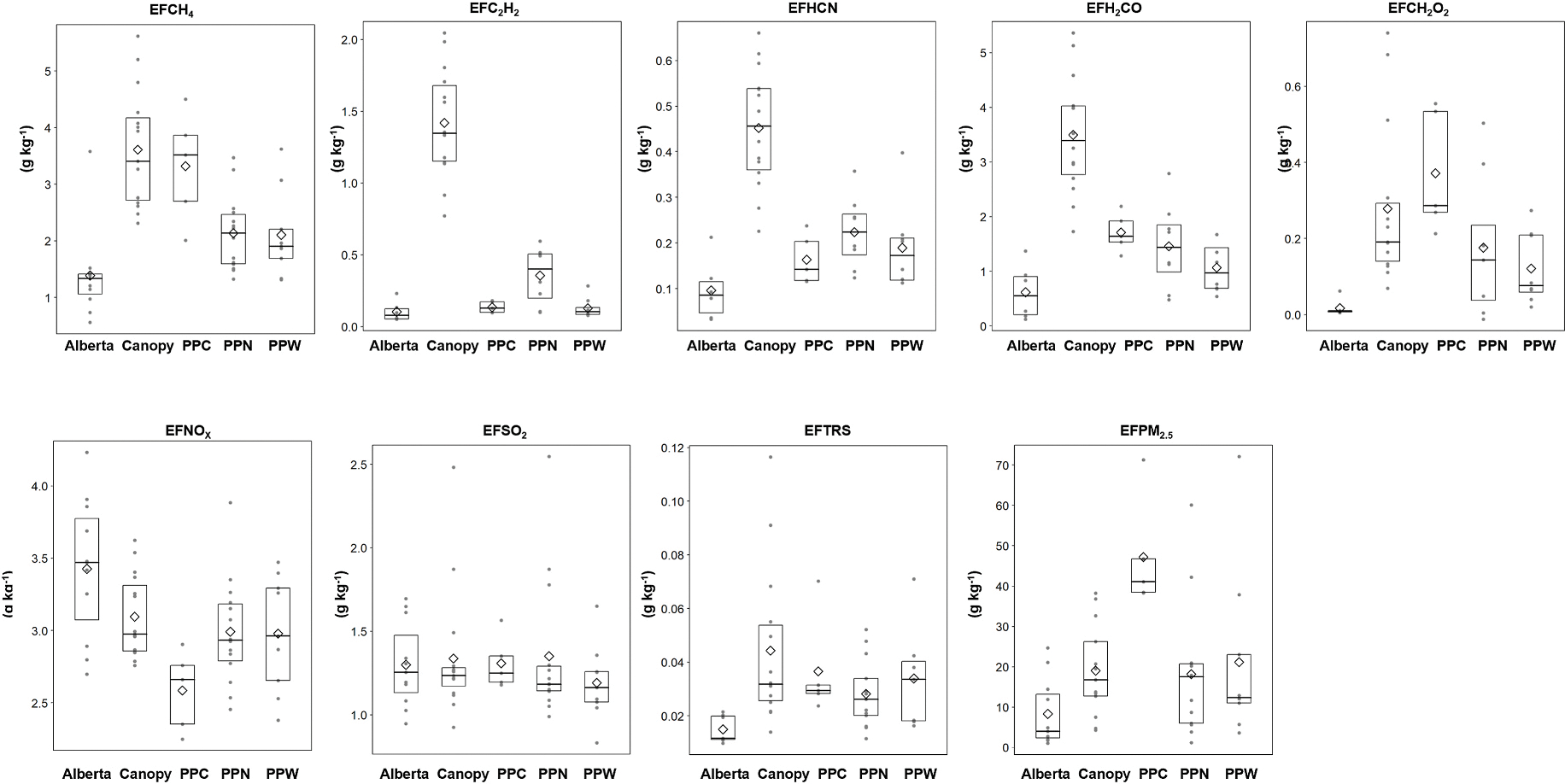
Emission factors by fuel type with median (thick black line), upper and lower quartiles (box), observations (gray filled circles) and mean (open diamond). Fuel type: Alberta = black spruce/jack pine litter and FWD, Canopy = Douglas fir canopy, PPN = ponderosa pine needles, PPW = ponderosa pine wood and needles, PPC = ponderosa pine cones and needles.

Excess mixing ratio time series for a PPN + PPW burn is shown in Fig. 2. EFs derived from the excess mixing ratio time series are summarized by fuel type in Table 2 and Fig. 3. Results for individual burns are provided in Appendix Table A.3. The Wilcoxon test was used to calculate pairwise comparisons of EFs between fuel types (Appendix Table A.4). The EFs for carbonaceous species (other than CO and CO_2_) were consistently lowest for the Alberta fuels and highest for the canopy fuels, with the exception of EFCH_2_O_2_ and EFPM_2.5_ for which PPN + PPC had the highest average value. Canopy EFs for CH_4_, C_2_H_2,_ HCN, and H_2_CO were significantly different from those of other fuels, except CH_4_ for PPN + PPC, see Appendix Table A.4. Canopy fuel EF ratios relative to the other fuel types (EF_canopy_/EF_other_) range over 4–14 for C_2_H_2_ and 2–6 for H_2_CO and HCN, respectively.

EFs for many compounds are correlated with MCE and the index has long been used to help explain EF variability within and across fuel types (Yokelson et al., 1999; Urbanski, 2014). Fig. 4 presents scatter plots of EFs *versus* MCE along with Pearson’s product-moment correlation coefficients and p-values for the best fit least square linear regression model. Detailed statistics for EF versus MCE comparisons are provided in Appendix Table A.5. In our study, MCE appears to explain some of the EF variability for all carbonaceous gases and TRS, particularly for CH_4_, H_2_CO and HCN (r^2^ ≥ 0.73). In addition to the global comparison shown in Fig. 4, we also tested for EF – MCE correlation for the Alberta, canopy, and PPN fuel types and these results are included in Appendix Table A.5. The PPN + PPW and PPN + PPC fuel types were not assessed individually due to the low number of burns with TILDAS data (Table 2).

Study average EFNO_X_ and EFSO_2_ are similar across all fuel types (Fig. 3) and Wilcoxon test showed that EFs for NO_x_ and SO_2_ were not significantly different between fuel types (Appendix Table A.4). The EFSO_2_ similarity is interesting considering the Alberta fuels had a much higher total sulfur content than the Montana fuels: Alberta = 0.08–0.15, ponderosa pine fuels: 0.03–0.06, and canopy ~0.07 (% dry mass), see Appendix Table A.2. The trend in study average EFTRS across fuel types is similar to that for carbonaceous species with Alberta fuels lowest (EFTRS = 0.015 g kg^−1^) and canopy fuels highest (EFTRS = 0.044 g kg^−1^). However, our analysis found differences in EFTRS were only significant when comparing Alberta and canopy fuels (Appendix Table A.4).

The average EFs were similar across the ponderosa pine fuelbeds with the notable exception of EFCH_4_, which was 60% higher for the PPN + PPC (3.32 ± 0.98) compared with the PPN (2.14 ± 0.66) and PPN + PPW (2.11 ± 0.77). However, despite this large difference in average EF, this fuel type difference is not statistically significant (Appendix Table A.4). Given the high burn to burn variability in EFCH_4_, five PPN + PPC burns is likely insufficient to robustly quantify possible differences relative to other fuel types and additional tests are needed. We believe this to be the first report of EFs for ponderosa pine cones. Since cones can be an important fuel component of ponderosa pine litter layers (Fonda and Varner, 2004), additional testing should be conducted to better quantify possible EFCH_4_ differences which may have important implications for CH_4_ emissions as discussed below.

Study average EFPM_2.5_ spanned a factor of five, being lowest for the Alberta fuels (8.3 g kg^−1^) and highest for PPN + PPC (47.2 g kg^−1^). In the midrange were EFPM_2.5_ for the PPN, PPN + PPW, and canopy, which had similar study averages of around 20 g kg^−1^ (Table 2). Wilcoxon rank sum test pairwise comparisons indicate the canopy *versus* PPN + PPC and canopy *versus* Alberta differences are statistically significant (Appendix Table A.4). In general, EFPM_2.5_ increased with decreasing MCE as expected (Fig. 4i) although the overall relationship was somewhat weak with a coefficient of determination (r^2^) = 0.24 (see Appendix Table A.5). PPN + PPC EFPM_2.5_ were all consistently above the EFPM_2.5_ – MCE trend line (Fig. 4i). Among the remaining fuelbeds there were a handful of burns with EFPM_2.5_ well above the linear regression model fit line.

**Fig. 5.**
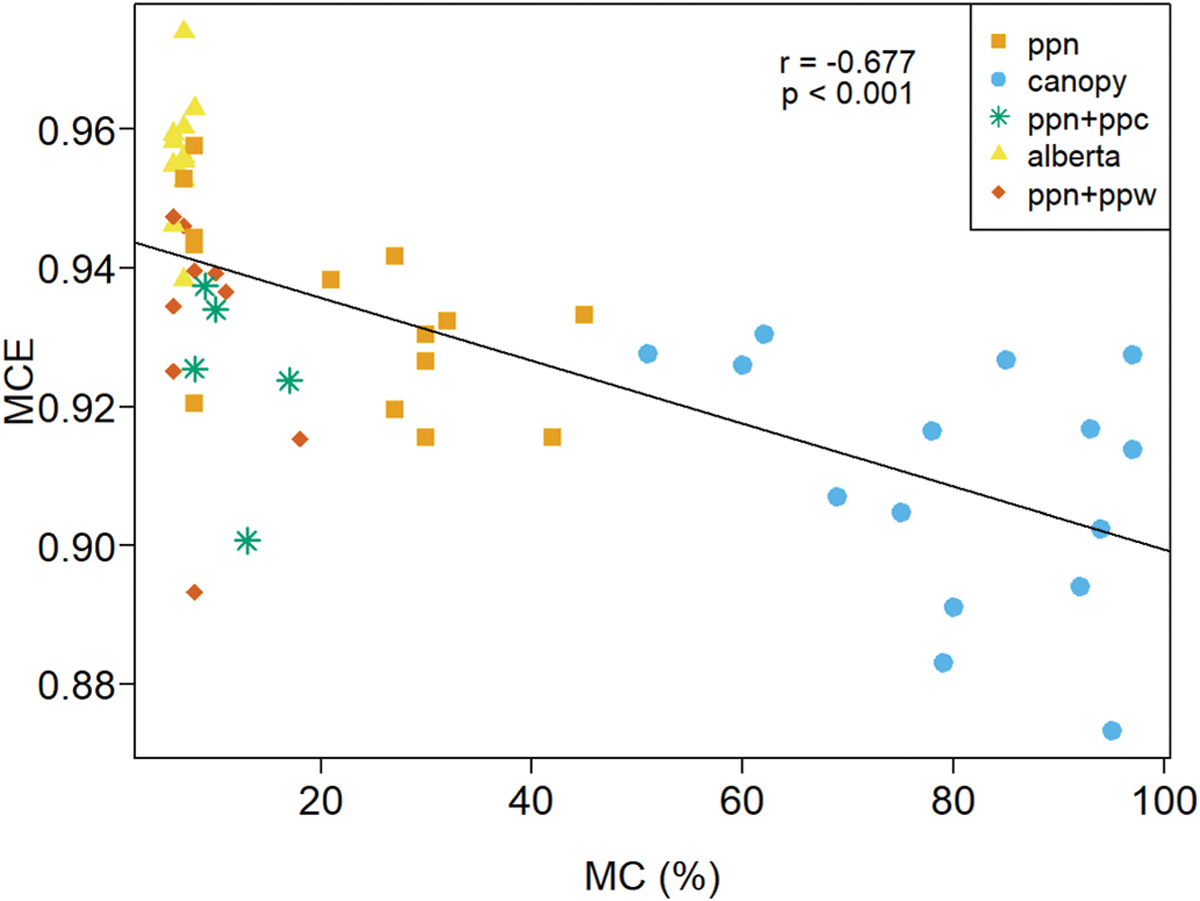
MCE plotted versus fuelbed moisture content (MC). Pearson’s product-moment correlation: r = −0.677 and p-value < 0.0001.

Fuel moisture content (MC) has a strong influence on flammability, fire spread rate, and overall fire behavior. With respect to emissions, increasing MC tends to increase smoldering combustion, decrease MCE, and increase EFs of non-CO_2_ carbonaceous pollutants (McMeeking et al., 2009). Our study’s fuelbed MC covered a wide range, being highest for the canopy (80 ± 15%) and lowest for the Alberta fuels (7 ± 1%) (see Table 2). The MC of needles, FWD, and cones used in our experiments are representative of typical real-world conditions during the wildfire season and spring prescribed burning season of the western U.S. and western Canada. However, the canopy fuels had an MC below that of natural conditions during the western U.S. wildfire season (MC > 100%). On a per burn basis, we observed an overall decrease in MCE with increasing MC (r = −0.677, p≤0.0001), see Fig. 5. MCE of the Alberta, PPN + PPW, and PPN + PPC fuelbeds were highly variable over a narrow MC range indicating other factors played an important role in the combustion process and emissions. The PPN and canopy burns each covered a wide range MC and offer the best opportunity to discern its influence on emissions. For the canopy burns, EFs for the volatile organic compunds (VOCs) and TRS were well correlated with MCE (Appendix Table A.5), however there was little correlation between MCE and MC (Pearson’s product-moment correlation r = −0.39, p = 0.15) suggesting MC was not a primary factor for emissions of these species. We observed no correlation between MC and any EFs for the canopy burns. The PPN EFs for the VOCs, TRS, and PM_2.5_ were well correlated with MCE (Appendix Table A.5) and the accompanying correlation between MC and MCE (Pearson’s product-moment correlation r = −0.60, p = 0.024) suggests MC was a contributing factor. In general, the MCE *versus* MC behavior we observed (Fig. 5) is similar to that observed by McMeeking et al. (2009) (their Fig. 4) – in particular high variability in MCE at MC < 10%.

### 3.2. Comparison with previous studies

The primary purpose of our study was addressing gaps in the EF literature related to Douglas fir canopy fuels and black spruce/jack pine and ponderosa pine litter and FWD. However, it is useful to compare our results with previous laboratory studies reporting EF for other fuel layers of these forest types. This comparison will help reveal the relevance of EFs measured in our study with respect to potential prescribed fire/wildfire emissions differences.

#### 3.2.1. Douglas fir canopy

**Fig. 6.**
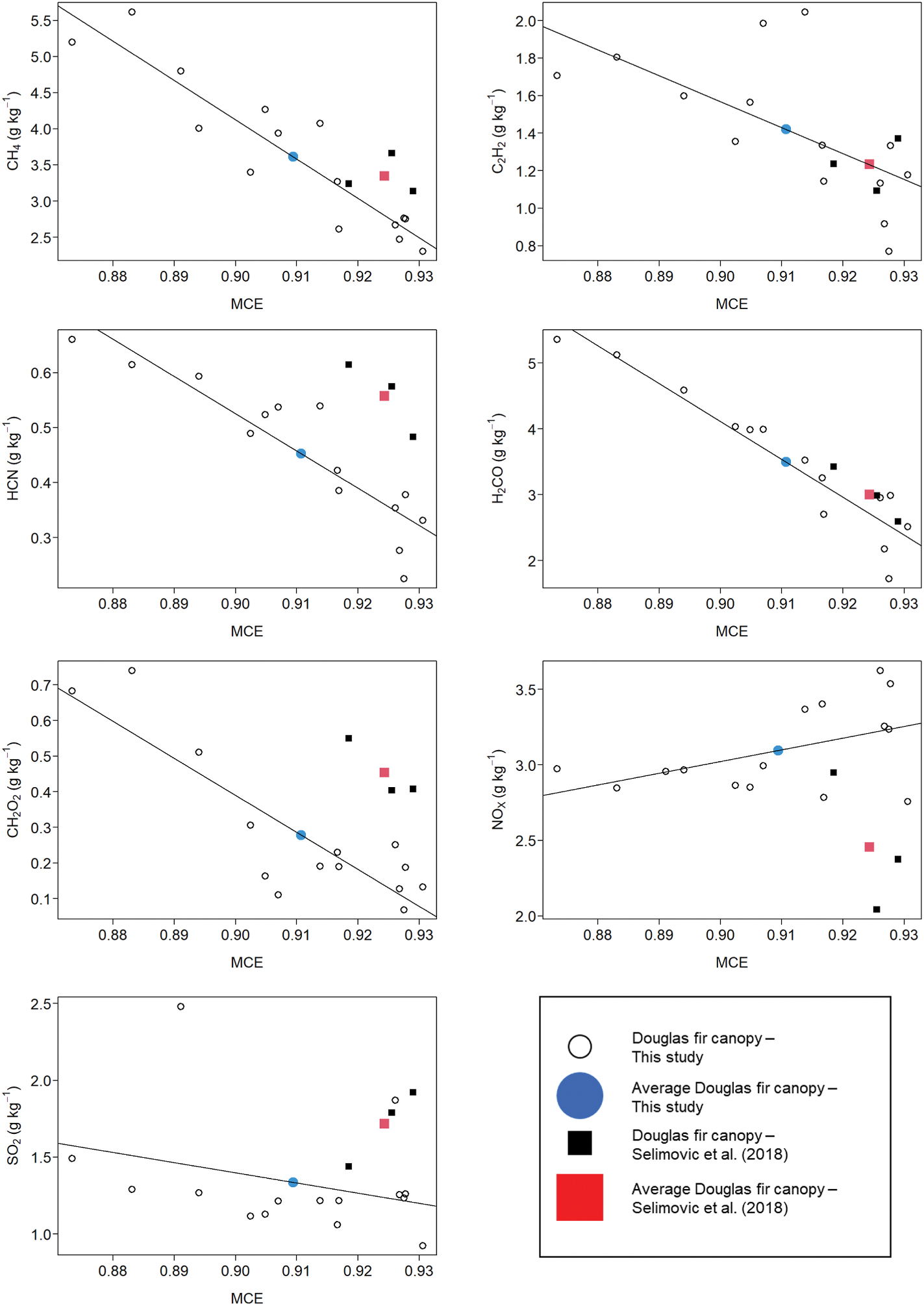
Plots of EF versus MCE for Douglas fir fuels from this study and Selimovic et al. (2018). The solid lines in Fig. 6 are a linear least square model fit to our canopy EFs, see Appendix Table A.5.

To the best of our knowledge, the only published EFs for Douglas fir canopy fuels are three burns provided in the supplemental material of Selimovic et al. (2018), one of several papers based on the National Oceanic and Atmospheric Administration Fire Influence on Regional and Global Environments Experiment (FIREX) laboratory intensive study. Selimovic et al. (2018) also reported EFs for Douglas fir fuel mixtures of litter, FWD, duff, and canopy fuels (their Table 1) and litter only (their supplemental material). Our comparison begins with their canopy EFs. The larger sample size for Douglas fir canopy burns in our study (n = 15) allowed us to measure emissions over a wider range of combustion conditions (MCE: 0.873–0.931 versus 0.918–0.929) and thus provide an improved characterization of the natural variability in EF for the compounds reported here. Fig. 6 plots EF *versus* MCE for both studies. (Note: the solid lines in Fig. 6 are a linear least squares fit to our canopy EFs, see Appendix Table A.5). The Selimovic et al. (2018) EFs for CH_4_, C_2_H_2_, and H_2_CO are in good agreement with the values we report, while their EFHCN and EFCH_2_O_2_ are somewhat higher. Our study average EFNOx was 30% higher than that of Selimovic et al. (2018) 3.10 ± 0.29 *versus* 2.46 ± 0.46, a difference that cannot be attributed to MCE (we found no correlation between EFNOx and MCE, see Appendix Table A.5) or fuel nitrogen content (0.91% in our study *versus* 1.01% Selimovic et al. (2018). EFSO_2_ were in rough agreement between the two studies (1.34 ± 0.38 *versus* 1.72 ± 0.25). Another study from the FIREX experiments, Koss et al. (2018), reports EFs based on proton transfer reaction time-of-flight mass spectroscopy (PTR-ToF MS) measurements. Koss et al. (2018) includes (in their supplemental material) EFs for 21 S-containing compounds (SO_2_ not included) averaged over 10 burns of Douglas fir fuel mixtures and individual components (litter, FWD, canopy, duff). While the aggregated nature of their data does not provide a direct comparison with our canopy results, it is worth noting the sum of their sulfur compounds EFs, 0.05 g-S kg^−1^, is comparable to the 0.044 g-S kg^−1^ we measured for EFTRS (Table 2).

Compared with the fuel mixture burns and litter, our canopy burns had lower MCE, higher EFCO, and higher VOC EFs, except for CH_2_O_2_ (Fig. 6). Our EFH_2_CO, EFHCN, EFC_2_H_2_ were more than twice that measured by Selimovic et al. (2018) for the fuel mixtures and litter and these differences track MCE (Fig. 6). Interestingly, EFC_2_H_2_ for the fuel mixture and litter are well below the EFC_2_H_2_ *versus* MCE trend, similar to the behavior of ponderosa pine and Alberta fuels in our study (Figs. 3 and 4).

#### 3.2.2. Black spruce and jack pine surface fuels

We believe the EFs reported in this study for the black spruce and jack pine litter and FWD (Alberta fuels) are the first to appear in the literature. Previous studies of boreal forest fuels have focused on spruce peat, with black spruce canopy fuels also receiving attention. Table 3 compares EFs from our Alberta fuels with those from previous studies of peat and canopy fuels. Boreal peatlands have garnered most of the attention as they are a tremendous store of carbon, estimated as 415 Gt (Beaulne et al., 2021), fires in this ecosystem are a large global source of pollutant emissions (van der Werf et al., 2017; Hu et al., 2018), and the warming boreal climate is expected to result in increased peatland fire activity and severity in the future. Peat burns largely by smoldering combustion (Hu et al., 2018) and as expected the EFs for incomplete combustion products CO, CH_4_, C_2_H_2_, HCN, H_2_CO, and CH_2_O_2_ are considerably higher than those measured for litter and FWD in our study (Table 3). While canopy fuels burn predominantly by flaming combustion, the VOC EFs reported for fresh black spruce canopy are considerably larger (by factors of 1.6–13.9) than those measured in our study, despite similar MCE (Table 3). This difference may be partially attributable to fuel moisture content, fresh coniferous canopy fuels typically have a high moisture content (see Appendix Table A1) and the litter and FWD burned in our study had moisture content <10% (Table A1). The sum of NO_x_ EFs for the litter + FWD (our study) and black spruce canopy are similar.

#### 3.2.3. Ponderosa pine surface fuels

Despite the importance of the ponderosa pine as a fire-dependent ecosystem in the western U.S., published EFs for the compounds measured in our study are limited. We compared our ponderosa pine burn EFs with those of Selimovic et al. (2018) for ponderosa pine fuel mixtures of litter, FWD, duff, and canopy fuels (their Table 1) and found agreement within roughly 50% for all compounds except NO and C_2_H_2_. Interestingly, their EFCH_4_ (2.76 ± 0.85 g kg^−1^) falls in nearly midrange between our EFCH_4_ values for PPN + PPC EFCH_4_ (3.55 ± 1.09 g kg^−1^) and our PPN and PPN + PPW (2.19 ± 0.88 and 2.29 ± 0.91 g kg^−1^, respectively) fuelbeds. We do not believe this observation contradicts our assertion that PPN + PPC emit more CH_4_ than PPN + PPW. The Selimovic et al. (2018) EFCH_4_ includes the contribution of duff; EFCH_4_ are high for duff and peat (e.g., Table 3 and Akagi et al., 2011; Urbanski 2014) and do not correlate well with MCE (Stockwell et al., 2016; Smith et al., 2018).

#### 3.2.4. PM_2.5_

**Fig. 7.**
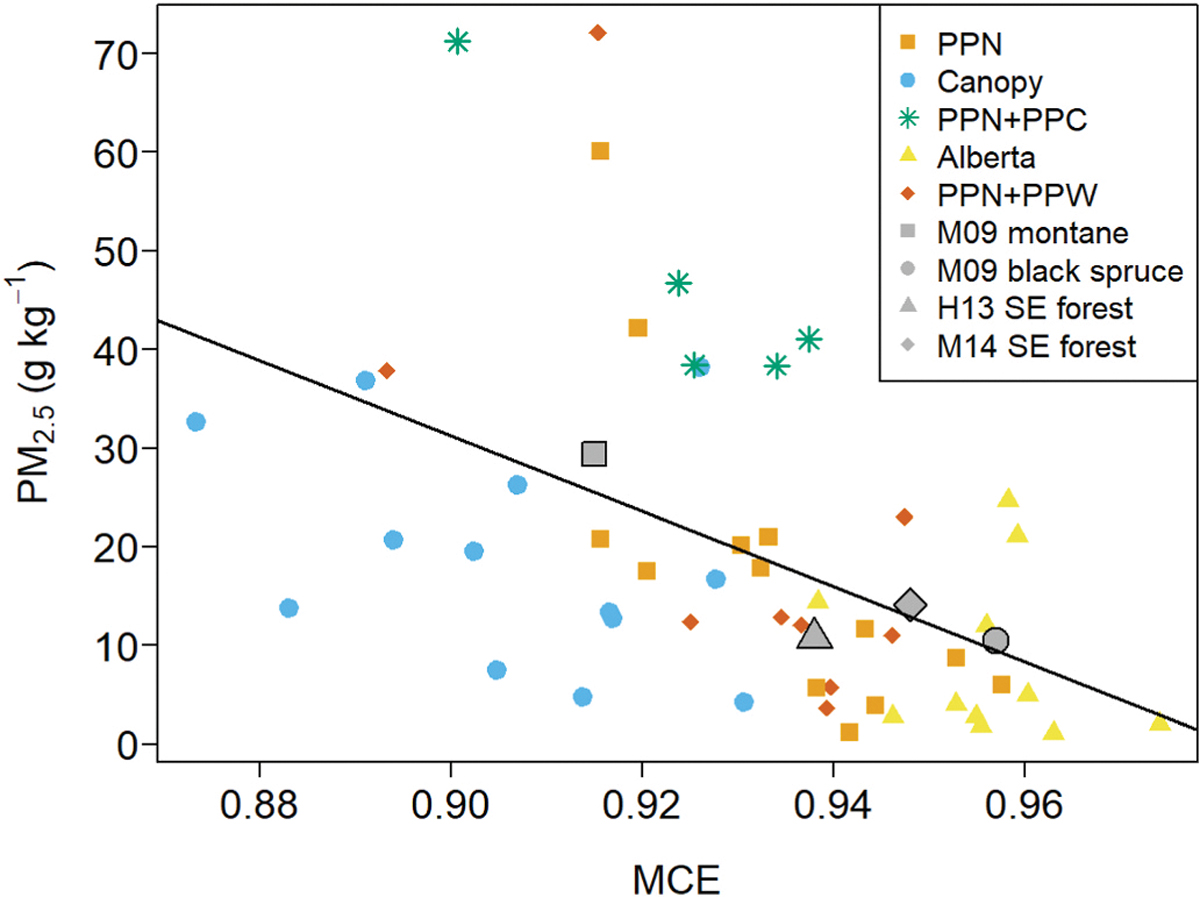
EFPM_2.5_ plotted versus MCE for our study – Alberta, Canopy, PPN, PPN + PPC, and PPN + PPW; McMeeking et al. (2009) – M09; Hosseini et al. (2013) – H13; and May et al. (2014) – M14. Solid gray line is linear least squares fit to our EFPM_2.5_ data, see Appendix Table A.5.

In Fig. 7 we compare our EFPM_2.5_ with study average values reported in three previous laboratory studies: McMeeking et al. (2009), Hosseini et al. (2013), and May et al. (2014). The EFPM_2.5_ from the previous studies are for a variety of fuels - western U.S. montane forests, black spruce, and southeastern U.S. forests and fall close to our EFPM_2.5_ versus MCE linear regression line. We note that May et al. (2014) report PM_1_, (particulate matter with aerodynamic diameter of <1 μm), however since the PM_2.5_ mass in fresh biomass smoke is concentrated in submicron particles (Reid et al., 2005b) PM_1_ and PM_2.5_ will be roughly equivalent. The black spruce results of McMeeking et al. (2009; EFPM_2.5_ = 10.4 ± 4.20 g kg^−1^ for MCE = 0.957 ± 0.012) are in excellent agreement with our results for Alberta fuels (EFPM_2.5_ = 8.3 ± 8.4 g kg^−1^ for MCE = 0.956 ± 0.009, Table 2). At 29.4 ± 25.1 g kg^−1^, the McMeeking et al. (2009) EFPM_2.5_ for montane fuels (needles and branch wood of ponderosa pine and lodgepole pine) were slightly higher than our PPN and PPN + PPW results (Table 2) and this likely reflects the lower MCE of the earlier study (0.915 ± 0.033 versus 0.933, see Table 2). Not shown in Fig. 7 are the results of May et al. (2104) for western U.S. montane forest fuels (ponderosa pine and lodgepole pine needles and branches). Over five laboratory burns May et al. (2014) measured EFPM_1_ = 167.1 ± 58.9 g kg^−1^ for MCE = 0.891 ± 0.017 which greatly exceeds the values we measured for similar fuels (PPN and PPN + PPW, Table 2) and is well above that predicted by our MCE-based regression: 34.7 g kg^−1^ at MCE = 0.891. The large difference may reflect the comparatively high MC of the May et al. (2014) burns, MC = 46%–83% *versus* 7%–45% for our PPN and PPN + PPW. However, we observed no correlation between EFPM_2.5_ and MC in our study and none was reported by May et al. (2014).

PM concentration was cited by May et al. (2014) as a likely factor behind the high EFPM_1_ measured in their study. PM_2.5_ emitted by biomass burning is largely organic aerosol (OA) by mass (McMeeking et al., 2009; Hosseini et al., 2013; May et al., 2014). The majority of these OA emissions are semi-volatile - the organic matter present in the particle phase can vary depending on ambient conditions with highly concentrated emissions favoring partitioning to the particle phase (May et al., 2013). In May et al. (2014) burn average OA concentrations ranged over 3160–6770 μg m^−3^ for the five montane forest fuel burns with EFPM_1_ = 167.1 ± 58.9 g kg^−1^. We did not measure OA in our study, but assuming 90% of PM_2.5_ was OA (McMeeking et al., 2009), the burn average OA concentrations for PPN and PPN + PPW fuels ranged over 60–2000 μg m^−3^ with an average of 720 μg m^−3^. Thus, the large difference in EFPM between our study and May et al. (2014) may be partially attributable to the studies’ very different OA concentrations.

#### 3.2.5. Implications

The effects of fire suppression on fuels under a changing climate are leading to an increasingly favorable environment for crown fire across seasonally dry conifer forest of the western U.S. and Canada. The primary objective of prescribed fire treatments in these forests is usually to modify fuel profiles to reduce crown fire potential (Agee and Skinner, 2005). Therefore, canopy fuels are a latent emission source that is potentially reduced by prescribed fire. In Douglas fir forests, canopy fuels account for ~20% of the total forest fuel load that is available for burning (Urbanski et al., 2018). The Douglas fir canopy EFs reported in this study can be used to quantify these potential emission reductions in assessments of land management strategies.

In ponderosa pine forests the litter layer accounts for ~60% of fuel loading typically targeted for removal with prescribed fire (Progar et al., 2017; Urbanski et al., 2018). The EFs we have measured for needles (the primary component of ponderosa pine litter layers), needles + FWD, and needles + cones can be used to improve prescribed fire emission estimates in smoke management tools such as First Order Fire Effects Model (FOFEM; Lutes, 2019). Our ponderosa pine cone EFs address a potentially important gap in the literature. Litter layers in fire stable pine forests, such as ponderosa pine, are heavily populated with fallen cones (Fonda and Varner, 2004). Pine cones are an important fuel component due to their long duration of smoldering and have been identified as an important smoke management concern. (Fonda and Varner, 2004).

Given the increased attention CH_4_ is receiving as a GHG emissions reduction target, our ponderosa pine EFCH_4_ may be of particular interest for management scenarios that consider GHGs. The EFCH_4_ for fuelbeds with cones was 60% higher than for those without (Table 2). However, with only five pine cones burns, the Wilcoxon test showed the difference between the fuel types was not statistically significant (Appendix Table A.4), despite this large difference in average EF. Additional testing should be conducted to better quantify possible EFCH_4_ differences. Since cones are not characterized as a separate component in fuel loading inventories but are subsumed into the litter layer (Woodall et al., 2019), we cannot estimate the additional CH_4_ emissions that might arise from smoldering cones for a typical prescribed fire. However, ponderosa pine are masting trees - they have periodic, non-cyclical heavy cone production years (mast years) followed by multiple years of comparatively low cone production (Shepperd et al., 2006; Keyes and Manso Gonzales, 2015) - therefore, temporal distancing of prescribed burns from mast years may be a strategy to consider if limiting CH_4_ emissions is a concern.

## 4. Conclusions

We have measured EFs for 1) EPA criteria pollutants CO, NO_X_, SO_2_, and PM_2.5_, 2) O_3_ precursors C_2_H_2_ and H_2_CO, 3) GHGs CO_2_ and CH_4_, and 4) HCN, an atmospheric tracer of biomass burning. This study has addressed two important gaps in the biomass burning EF literature – Douglas fir canopy fuels and black spruce/jack pine surface fuels. These forest types are a significant portion of forests consumed by wildfires in western U.S. and Canada, a region experiencing significant increases in burned area and pollutant emissions. This study also examines the variability of pollutant emissions across components of ponderosa pine forest surface fuelbeds: needles, fine woody debris, and cones. While emissions from ponderosa pine surface fuels have been studied previously, our study is the first to quantify EFs for cones, an important component of the litter layer. We also found that EFTRS was correlated with MCE, EFSO_2_ was not correlated with MCE, and that a factor of 2 difference in fuel S content did not have a discernible effect on emission of either reduced (TRS) or oxidized (SO_2_) emissions. Most importantly, our EF measurements provide fuel layer specific data that is needed to evaluate the emission consequences of different land management strategies for addressing increased fire activity, declining forest health, and the growing threat to life and property from an expanding urban – wildland interface.

## Figures and Tables

**Fig. 4a-e.**
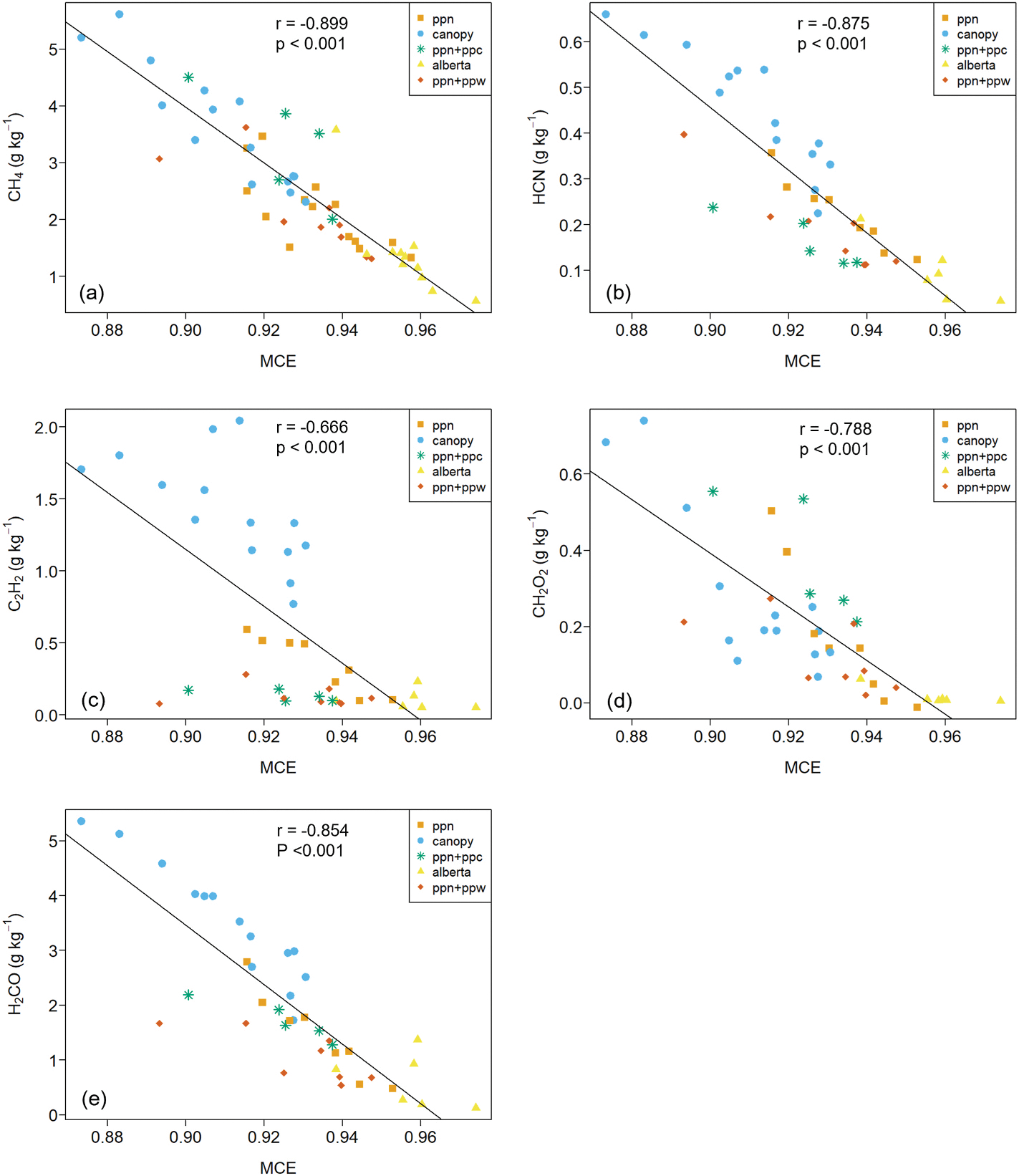
EF plotted versus MCE for all 54 burns. Alberta = black spruce/jack pine litter and FWD, canopy = Douglas fir canopy, PPN = ponderosa pine needles, PPN + PPW = ponderosa pine needles and FWD, PPN + PPC = ponderosa pine needles and cones. Complete statistics for EF *versus* MCE linear egressions are given in Appendix Table A.5.

**Fig. 4f-i.**
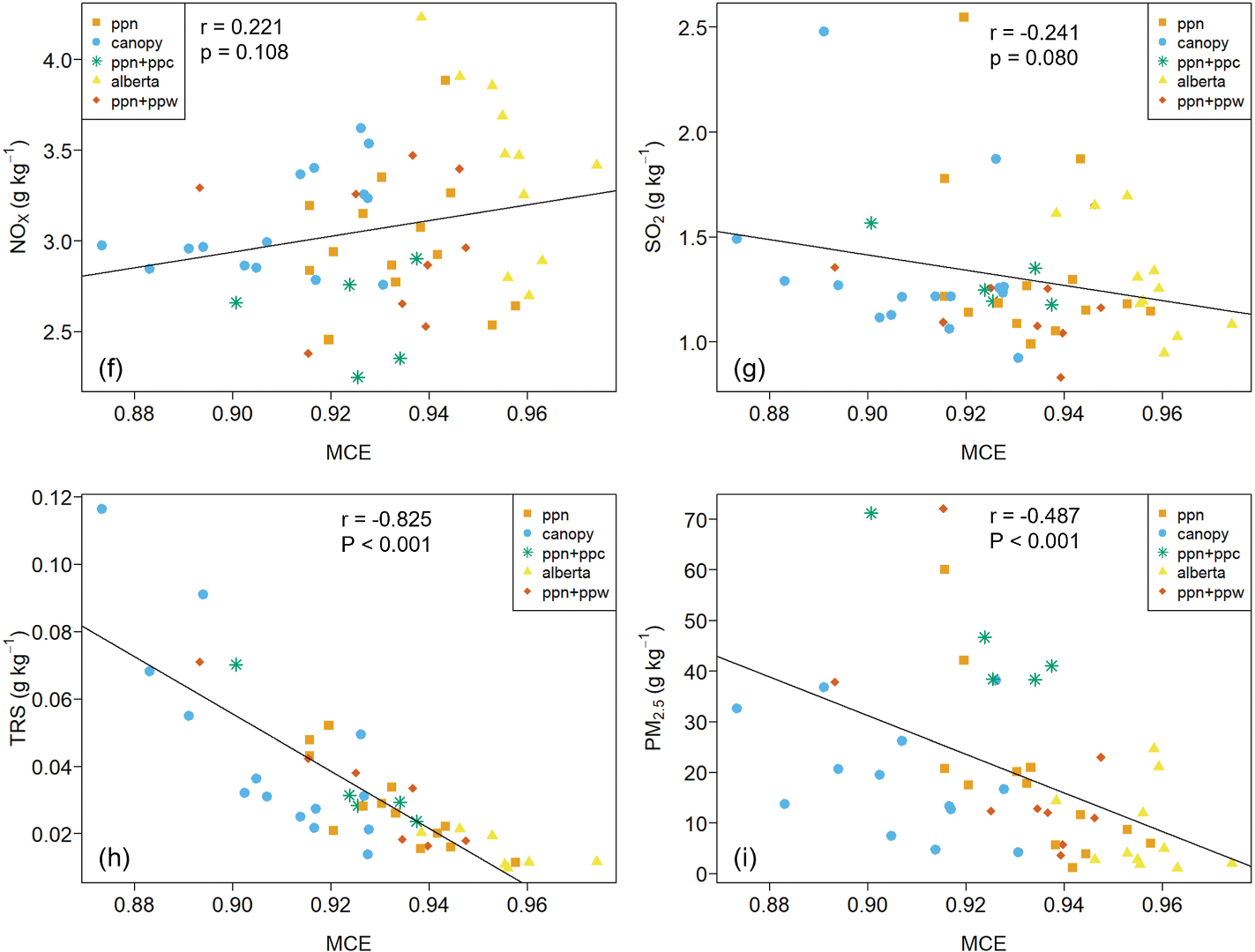
EF plotted versus MCE for all 54 burns. Alberta = black spruce/jack pine litter and FWD, canopy = Douglas fir canopy, PPN = ponderosa pine needles, PPN + PPW = ponderosa pine needles and FWD, PPN + PPC = ponderosa pine needles and cones. Complete statistics for EF *versus* MCE linear regressions are given in Appendix Table A.5.

**Table 1:** Instrument details.

Pollutant	Manufacturer	Model	Method	FRM/FEM

CO,CO_2_,CH_4_	Piccarro	G2401-m	CRDS^a^	NA
NO	Teledyne API	T200U	CL (O_3_)^b^	FRM
NO_2_	Teledyne API	T500U	CAPS^c^	FEM
PM_2.5_	TSI	3321	APS^d^	NA
TRS	Teledyne API	T102	UVf^e^	NA
SO_2_	Thermo Scientific	43C	UVf^e^	FEM
C_2_H_2_, H_2_CO, CH_2_O_2_, HCN	Aerodyne		TILDAS^f^	NA
CO_2_	LI-COR	LI-850	NDIR^g^	NA

All analyzers except for the LI-850 were operated on the stack platform and sampled directly from the stack through short (~1.5 m) lengths of 6.4 mm PFA tubing. The LI-850 was operated on the chamber floor to quantify background concentrations of CO_2_.

a Cavity Ring-Down Spectroscopy.

b Ozone Chemiluminescence.

c Cavity Atennuated Phase Shift Spectroscopy.

d Aerosol Particle Sizer-Time-Of-Flight Dual Laser Spectrometer.

e UV Fluorescence.

f Tunable Infrared Laser Direct Absorption Spectroscopy.

g Non-Dispersive Infrared Absorption.

**Table 2:** Average emission factors (g/kg) by fuel type.

	Ponderosa pine needles	Ponderosa pine needles and FWD^a^	Ponderosa pine needles and cones	Douglas fir canopy^b^	Jack pine and black spruce^c^

N	14	9	5	15	11
N TILDAS	8	8	5	14	6
N TRS	13	7	5	14	7
Moisture Content (%)	23(13)	9(4)	11(4)	80(15)	7(1)
MCE	0.934 (0.013)	0.931 (0.017)	0.924 (0.014)	0.909 (0.018)	0.956 (0.009)
CO_2_	1721(60)	1732(80)	1632(58)	1666 (56)	1818 (36)
CO	78(14)	81(18)	85(14)	105(20)	53(11)
CH_4_	2.14(0.66)	2.11(0.77)	3.32(0.98)	3.61(1.05)	1.39(0.79)
C_2_H_2_^d^	0.357(0.196)	0.13(0.071)	0.137(0.039)	1.42(0.383)	0.105(0.07)
HCN^d^	0.224(0.078)	0.189(0.095)	0.164(0.055)	0.452(0.133)	0.096(0.067)
H_2_CO^d^	1.458(0.781)	1.069(0.46)	1.714(0.35)	3.496(1.081)	0.62(0.503)
CH_2_O_2_^d^	0.176(0.185)	0.121(0.095)	0.371(0.161)	0.278(0.213)	0.017(0.022)
NO	2.088(0.383)	2.163(0.352)	1.867(0.276)	2.265(0.268)	2.696(0.428)
NO_2_	1.387(0.346)	1.252(0.132)	1.101(0.127)	1.275(0.234)	1.121(0.276)
NO_x_	2.993(0.371)	2.98(0.399)	2.585(0.276)	3.096(0.285)	3.427(0.488)
SO_2_	1.351(0.428)	1.192(0.23)	1.309(0.159)	1.337(0.38)	1.299(0.255)
TRS	0.028(0.013)	0.034(0.019)	0.037(0.019)	0.044(0.029)	0.015(0.005)
SO_2_(as S)	0.676(0.214)	0.596(0.115)	0.654(0.08)	0.668(0.19)	0.65(0.128)
TRS/SO_2_	0.042	0.057	0.056	0.066	0.023
PM_2.5_	18.21(16.54)	21.2(21.66)	47.17(13.88)	19.05(11.49)	8.32(8.41)

Values in brackets are (1 σ) standard deviation.

a Fine woody debris (FWD).

b A small amount of Ponderosa pine needles were used to ignite each fire (see text).

c Needles and fine woody debris(FWD).

d Compounds measured via TILDAS.

**Table 3:** Emission factors (g kg^−1^) for black spruce/jack pine fuel components.

	Litter & FWD	Boreal Peat^b^			Canopy^c^
			
	This Study^a^	Watson et al. (2019)	Stockwell et al. (2014)	Yokelson et al. (1997)	Stockwell et al. (2014)

MCE^d^	0.956(0.009)	0.85(0.02)	0.805(0.009)	0.809(0.327)	0.951(0.012)
CO_2_	1818(36)	1400(38)	1274(19)	1395(52)	1737(28)
CO	53(11)	161(19)	197(9)	209(68)	57(13)
CH_4_	1.39(0.79)	5.69(1.07)	6.25(2.17)	6.85(5.65)	2.42(0.76)
C_2_H_2_	0.105(0.07)		0.10(0.00)	0.10(0.00)	0.75(0.28)
HCN	0.096(0.067)	2.33(0.22)	1.77(0.55)	5.09(5.64)	0.23(0.07)
H_2_CO	0.62(0.503)		1.43(0.37)	1.99(2.67)	1.35(0.45)
CH_2_O_2_	0.017(0.022)		0.40(0.06)	0.89(1.15)	0.25(0.13)
NO	2.696(0.428)	0.84(0.44)	–	–	1.97(0.32)
NO_2_	1.121(0.276)	0.37(0.13)	–	–	2.06(0.33)
SO_2_	1.299(0.255)				0.60(0.32)

Values in brackets are (1 σ) standard deviation.

a Needles and fine woody debris (FWD).

b Boreal peat from black spruce forests in Alaska, Canada, Minnesota.

c Black spruce canopy from Alaska.

d MCE (modified combustion efficiency) = ΔCO_2_/(ΔCO+ΔCO_2_).

**Appendix Fig. B.2.**
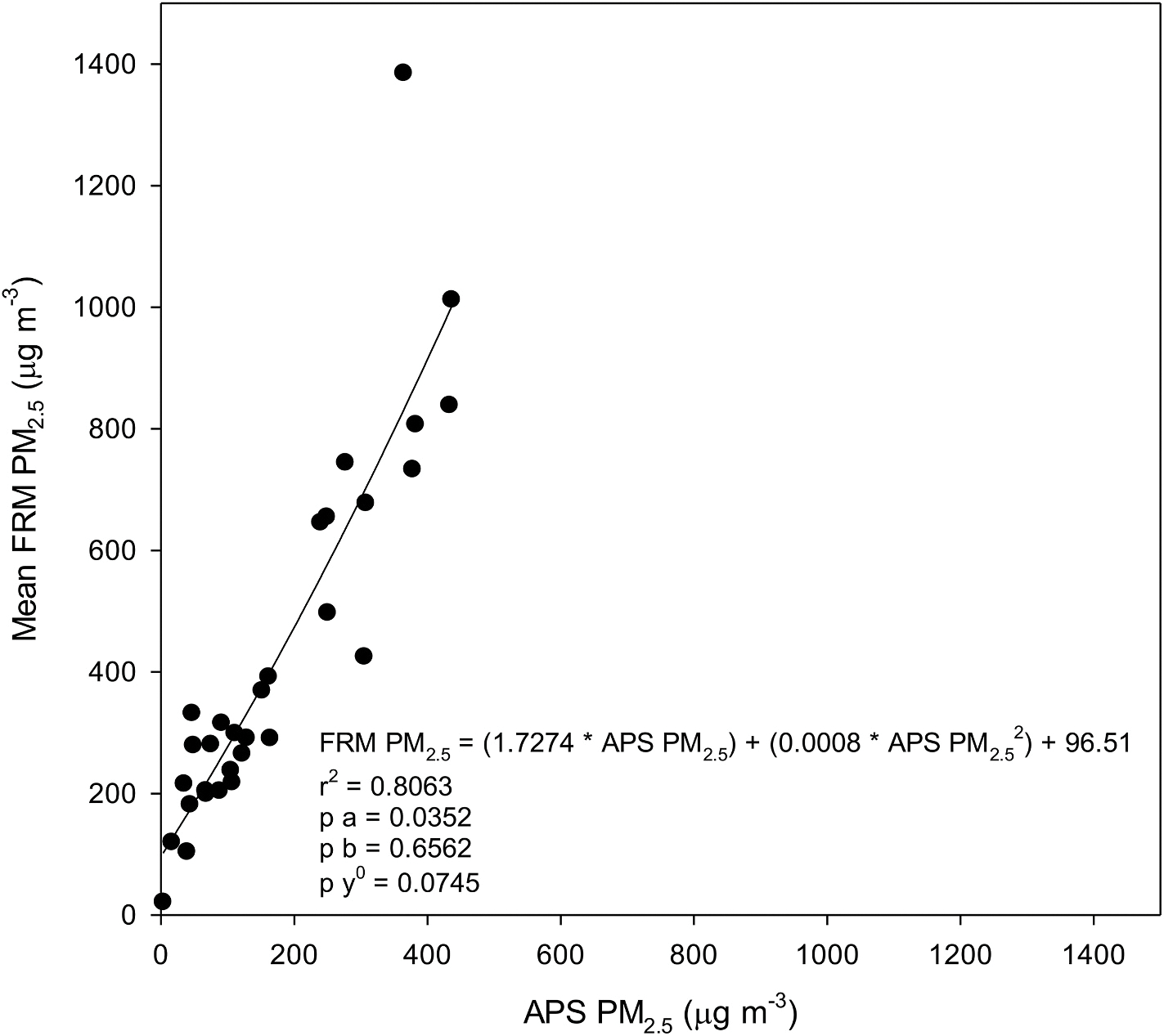
APS PM_2.5_ *versus* FRM PM_2.5_ Quadratic Model Calibration Equation.

## Data Availability

Datasets related to this article can be found at https://catalog.data.gov/dataset/epa-sciencehub.

## Appendix A. Supplementary data

### Appendix A Supplementary Tables

**Appendix Table A.1:** Fuels.

Burn	Needle (g)	Wood (g)	Other (g)	Needle moisture content (%)	Wood moisture content (%)	Other ^1^ moisture content (%)	Fuelbed ^2^ moisture content (%)	Post (g)

Ponderosa pine needles								
1	278	0	0	8	–	–	8	21
2	211	0	0	42	–	–	42	122
10	114	0	0	32	–	–	32	0
11	172	0	0	8	–	–	8	8
12	173	0	0	8	–	–	8	19
13	133	0	0	45	–	–	45	35
15	196	0	0	27	–	–	27	26
16	205	0	0	27	–	–	27	17
26	142	0	0	30	–	–	30	16
29	159	0	0	7	–	–	7	10
30	115	0	0	30	–	–	30	17
34	142	0	0	30	–	–	30	34
40	166	0	0	21	–	–	21	16
55	139	0	0	8	–	–	8	13
Ponderosa pine needles and FWD^3^								
5	97	160	0	8	7	–	7	155
14	171	69	0	8	7	–	8	72
18	141	60	0	8	7	–	8	19
36	163	140	0	9	11	–	10	41
43	147	130	0	6	7	–	6	82
44	166	92	0	6	7	–	6	53
46	84	45	0	6	7	–	6	20
51	165	82	0	18	17	–	18	19
57	163	111	0	13	8	–	11	97
Ponderosa pine needles and cones								
31	148	0	111	8	–	8	8	31
45	143	0	120	6	–	21	13	51
50	123	0	108	9	–	11	10	21
52	140	0	80	8	–	10	9	35
58	102	0	68	13	–	22	17	40
Douglas-fir canopy								
3	56	0	144	8	–	108	80	218
17	19	0	160	8	–	34	31	179
19	46	0	131	8	–	79	60	98
24	23	0	138	7	–	58	51	130
25	23	0	160	7	–	78	69	0
32	60	0	128	9	–	120	85	193
33	27	0	103	9	–	120	97	108
35	38	0	131	9	–	94	75	0
38	20	0	55	11	–	102	78	39
39	17	0	62	11	–	77	62	0
41	27	0	147	11	–	113	97	159
42	30	0	100	11	–	100	79	70
47	24	0	146	6	–	106	92	174
49	36	0	175	9	–	110	93	200
54	24	0	169	8	–	108	95	216
56	23	0	141	8	–	108	94	213
Black spruce and jack pine surface fuels (Alberta fuels)								
8	244	74	0	7	9	–	7	97
9	64	74	0	7	9	–	8	13
20	65	53	0	5	7	–	6	43
22	121	67	0	5	7	–	6	59
21	0	164	0	–	7	–	7	16
23	0	151	0	0	7	–	7	22
28	137	0	0	7	–	–	7	13
4	131	76	0	7	7	–	7	45
6	53	88	0	5	7	–	6	42
7	117	96	0	5	7	–	6	29
27	133	93	0	7	7	–	7	16

1 Douglas fir canopy or ponderosa pine cones.

2 Mass-weighted average of all fuelbed components.

3 fine woody debris (FWD).

**Appendix Table A.2:** Summary of Fuel Chemical Analysis.

Fuel species	Burn	Fuel type^1^	Sulfur (%DM)	Carbon (%DM)	Nitrogen (%DM)

Black spruce	Burns 20, 22	Needles	0.08	52.84	0.65
		FWD	0.09	52.16	0.69
	Burns 8, 9, 21, 23	Needles	0.15	51.26	1.01
		FWD	0.08	53.15	0.68
Jack pine	All burns	Needles	0.10	52.97	1.08
		FWD	0.10	51.55	0.73
Ponderosa pine	All burns	Needles	0.06	51.55	0.68
		FWD	0.03	53.49	0.45
		Cones	0.04	51.69	0.64
Douglas-fir	All burns	Canopy	0.07	51.57	0.91
		litter	0.06	52.60	0.90
		FWD	0.05	51.10	0.66
		cones	0.14	51.39	0.69

1 Fine woody debris (FWD).

**Appendix Table A.3:** Emission Factors (g kg^−1^).

Burn	MCE	CO_2_	CO	CH_4_	C2H2	HCN	H2CO	CH2O_2_	NO	NO_2_	NOX	SO_2_	TRS	SO_2_ (as S)	PM_2.5_

Ponderosa pine needles (PPN)															
1	0.943	1753	67	1.62	–	–	–	–	3.129	1.160	3.886	1.872	0.022	0.936	11.7
2	0.916	1679	98	2.51	–	–	–	–	2.020	1.800	3.194	1.778	0.043	0.889	20.8
10	0.932	1717	79	2.23	–	–	–	–	1.901	1.480	2.866	1.268	0.034	0.634	17.9
11	0.958	1794	51	1.33	–	–	–	–	2.148	0.757	2.642	1.147	0.012	0.573	6.0
12	0.921	1697	93	2.05	–	–	–	–	2.346	0.912	2.941	1.141	0.021	0.571	17.5
13	0.933	1711	78	2.57	–	–	–	–	1.845	1.424	2.773	0.991	0.026	0.495	21.0
15	0.942	1772	70	1.70	0.311	0.186	1.159	0.049	1.808	1.712	2.925	1.297	0.020	0.649	1.2
16	0.920	1631	91	3.47	0.517	0.283	2.050	0.396	1.626	1.271	2.456	2.547	0.052	1.274	42.2
26	0.930	1704	81	2.34	0.493	0.254	1.780	0.143	2.188	1.783	3.351	1.087	0.029	0.543	20.2
29	0.953	1777	56	1.59	0.106	0.124	0.480	−0.012	1.882	1.002	2.536	1.182	–	0.591	8.7
30	0.916	1582	93	3.26	0.595	0.357	2.791	0.504	1.741	1.681	2.837	1.216	0.048	0.608	60.1
34	0.927	1745	88	1.51	0.501	0.257	1.719	0.181	2.073	1.654	3.151	1.185	0.028	0.593	–
40	0.938	1754	73	2.27	0.230	0.193	1.128	0.143	2.011	1.632	3.075	1.052	0.016	0.526	5.7
55	0.944	1773	66	1.48	0.100	0.138	0.557	0.004	2.515	1.149	3.264	1.152	0.016	0.576	3.9
Ponderosa pine needles and fine woody debris (PPN + PPW)															
5	0.946	1802	65	1.34	–	–	–	–	2.53	1.33	3.40	1.65	–	0.83	11.0
14	0.893	1614	123	3.07	0.080	0.40	1.67	0.21	2.45	1.30	3.29	1.36	0.07	0.68	37.9
18	0.940	1779	73	1.69	0.079	0.11	0.54	0.02	2.17	1.07	2.87	1.04	0.02	0.52	5.8
36	0.939	1792	74	1.90	0.088	0.11	0.69	0.08	1.82	1.09	2.53	0.83	–	0.42	3.7
43	0.947	1765	62	1.31	0.117	0.12	0.68	0.04	2.12	1.29	2.96	1.16	0.02	0.58	23.0
44	0.935	1755	78	1.87	0.093	0.14	1.17	0.07	1.94	1.09	2.65	1.08	0.02	0.54	12.9
46	0.925	1738	90	1.96	0.116	0.21	0.77	0.07	2.37	1.37	3.26	1.26	0.04	0.63	12.4
51	0.915	1578	93	3.62	0.283	0.22	1.67	0.27	1.52	1.32	2.38	1.09	0.04	0.55	72.1
57	0.937	1762	76	2.21	0.183	0.20	1.35	0.21	2.55	1.42	3.47	1.25	0.03	0.63	12.0
Ponderosa pine needles and cones (PPN + PPC)															
31	0.925	1652	85	3.87	0.098	0.143	1.639	0.286	1.563	1.052	2.249	1.195	0.028	0.598	38.4
45	0.901	1533	108	4.50	0.173	0.238	2.191	0.555	1.963	1.071	2.661	1.567	0.070	0.783	71.3
50	0.934	1669	75	3.52	0.131	0.116	1.537	0.269	1.652	1.074	2.353	1.352	0.029	0.676	38.4
52	0.937	1673	71	2.01	0.100	0.118	1.284	0.213	2.260	0.986	2.903	1.179	0.024	0.590	41.1
58	0.924	1632	86	2.70	0.181	0.203	1.918	0.535	1.899	1.319	2.759	1.250	0.031	0.625	46.7
Douglas fir canopy															
3	0.891	1594	124	4.80	–	–	–	–	2.015	1.446	2.958	2.481	0.055	1.241	36.8
19	0.926	1650	84	2.67	1.135	0.354	2.958	0.252	2.774	1.305	3.625	1.873	0.050	0.936	38.2
24	0.928	1701	84	2.76	1.335	0.378	2.987	0.188	2.614	1.418	3.539	1.263	0.021	0.632	16.8
25	0.907	1635	107	3.94	1.985	0.537	3.993	0.111	2.148	1.297	2.994	1.215	0.031	0.607	26.2
32	0.927	1741	88	2.48	0.917	0.276	2.177	0.128	2.432	1.265	3.257	1.258	0.031	0.629	–
33	0.927	1743	87	2.76	0.772	0.226	1.727	0.069	2.269	1.484	3.237	1.235	0.014	0.618	–
35	0.905	1673	112	4.27	1.564	0.524	3.989	0.164	2.505	0.533	2.853	1.130	0.036	0.565	7.6
38	0.917	1686	98	3.27	1.336	0.423	3.257	0.230	2.482	1.414	3.405	1.063	0.022	0.531	13.4
39	0.931	1737	83	2.31	1.178	0.331	2.513	0.133	1.918	1.288	2.759	0.925	–	0.463	4.3
41	0.914	1696	102	4.08	2.046	0.539	3.525	0.191	2.432	1.436	3.368	1.218	0.025	0.609	4.7
42	0.883	1614	136	5.62	1.805	0.615	5.129	0.741	1.920	1.424	2.849	1.292	0.068	0.646	13.8
47	0.894	1624	123	4.01	1.598	0.594	4.589	0.511	2.138	1.271	2.967	1.271	0.091	0.635	20.7
49	0.917	1691	98	2.62	1.144	0.386	2.702	0.190	1.970	1.253	2.787	1.219	0.028	0.610	12.8
54	0.873	1556	144	5.20	1.707	0.661	5.362	0.684	2.293	1.046	2.975	1.492	0.117	0.746	32.7
56	0.902	1645	113	3.40	1.356	0.489	4.033	0.307	2.059	1.238	2.866	1.118	0.032	0.559	19.6
Black spruce and jack pine surface fuels (Alberta fuels)															
4	0.953	1820	57	1.43	–	–	–	–	3.205	1.000	3.858	1.696	0.019	0.848	4.0
6	0.946	1798	65	1.39	–	–	–	–	2.939	1.484	3.907	1.649	0.021	0.825	2.8
7	0.955	1823	55	1.41	–	–	–	–	3.069	0.952	3.689	1.307	–	0.654	2.8
8	0.956	1782	52	1.34	–	–	–	–	2.118	1.042	2.798	1.196	0.010	0.598	11.9
9	0.963	1843	45	0.74	–	–	–	–	2.375	0.792	2.892	1.026	–	0.513	1.1
20	0.959	1792	48	1.15	0.233	0.122	1.373	0.011	2.476	1.194	3.255	1.254	–	0.627	21.1
21	0.974	1893	32	0.56	0.053	0.033	0.124	0.005	2.862	0.853	3.418	1.084	0.012	0.542	2.0
22	0.958	1784	49	1.52	0.134	0.093	0.932	0.006	2.391	1.655	3.470	1.339	–	0.670	24.7
23	0.960	1859	49	0.98	0.053	0.036	0.186	0.007	2.108	0.904	2.697	0.947	0.011	0.474	4.9
27	0.955	1828	54	1.21	0.060	0.079	0.272	0.009	2.772	1.083	3.479	1.182	0.011	0.591	1.8
28	0.938	1779	74	3.58	0.097	0.213	0.830	0.062	3.341	1.368	4.233	1.612	0.020	0.806	14.4

1 MCE (modified combustion efficiency) = ΔCO_2_/(ΔCO+ΔCO_2_).

2 Fine woody debris (FWD).

**Appendix Table A.4:** Results for pairwise Wilcoxon rank sum hypothesis tests of EFs between fuel types.

EFCH_4_					EFNO_x_				
	Alberta	Canopy	PPC	PPN		Alberta	Canopy	PPC	PPN

Canopy	**0.000**	–	–	–	Canopy	0.869	–	–	–
PPC	**0.032**	1.000	–	–	PPC	0.055	0.059	–	–
PPN	**0.014**	**0.001**	0.194	–	PPN	0.287	1.000	0.339	–
PPW	0.159	**0.007**	0.290	1.000	PPW	0.465	1.000	1.000	1.000
									
EFC_2_H_2_					EFSO_2_				
	Alberta	Canopy	PPC	PPN		Alberta	Canopy	PPC	PPN
Canopy	**0.006**	–	–	–	Canopy	1.000	–	–	–
PPC	1.000	**0.002**	–	–	PPC	1.000	1.000	–	–
PPN	0.168	**0.000**	0.567	–	PPN	1.000	1.000	1.000	–
PPW	1.000	**0.000**	1.000	0.148	PPW	1.000	1.000	1.000	1.000
									
EFHCN	Alberta	Canopy	PPC	PPN	EFTRS TRS	Alberta	Canopy	PPC	PPN
Canopy	**0.001**	–	–	–	Canopy	0.008	–	–	–
PPC	1.000	**0.003**	–	–	PPC	0.057	1.000	–	–
PPN	0.080	**0.004**	1.000	–	PPN	0.078	0.987	1.000	–
PPW	0.609	**0.007**	1.000	1.000	PPW	0.547	1.000	1.000	1.000
									
EFH_2_CO	Alberta	Canopy	PPC	PPN	EFPM_2.5_	Alberta	Canopy	PPC	PPN
Canopy	**0.001**	–	–	–	Canopy	0.129	–	–	–
PPC	0.087	**0.012**	–	–	PPC	0.005	0.002	–	–
PPN	0.426	**0.002**	1.000	–	PPN	0.933	1.000	0.098	–
PPW	1.000	**0.000**	0.653	1.000	PPW	0.562	1.000	0.190	1.000
EFCH_2_O_2_									
	Alberta	Canopy	PPC	PPN					
Canopy	**0.001**	–	–	–					
PPC	**0.043**	1.000	–	–					
PPN	1.000	1.000	0.478	–					
PPW	**0.027**	0.502	0.062	1.000					

The table shows Bonferroni adjusted p-values. EF pairs with p < 0.05 are concluded to have different medians (level of significance is **α** = 0.05) and printed in **bold** typeface. Fuel types: Alberta = Jack pine and black spruce, Canopy = Douglas-fir canopy, PPC = Ponderosa pine needles and cones, PPN = Ponderosa pine needles, and PPW = Ponderosa pine needles and fine woody debris.

**Appendix Table A.5:** Emission Factor (EF) *versus* Modified Combustion Efficiency (MCE^1^).

	n	r	p-value	Intercept	Slope	r^2^

All burns						
EFCH_4_	54	−0.899	0.000	48.050	−48.969	0.808
EFC_2_H_2_	41	−0.666	0.000	18.957	−19.784	0.443
EFHCN	41	−0.875	0.000	6.627	−6.857	0.765
EFH_2_CO	41	−0.854	0.000	52.306	−54.267	0.729
EFCH_2_O_2_	41	−0.788	0.000	6.726	−7.036	0.620
EFNO_x_	54	0.221	0.108	−0.961	4.333	0.049
EFSO_2_	54	−0.241	0.080	4.694	−3.642	0.058
EFTRS	46	−0.825	0.000	0.820	−0.849	0.681
EFPM_2.5_	51	−0.487	0.000	374.572	−381.470	0.237
Douglas-fir canopy						
EFCH_4_	15	−0.926	0.000	53.027	−54.336	0.857
EFC_2_H_2_	14	−0.641	0.013	14.007	−13.820	0.411
EFHCN	14	−0.908	0.000	6.632	−6.786	0.825
EFH_2_CO	14	−0.945	0.000	55.924	−57.567	0.893
EFCH_2_O_2_	14	−0.868	0.000	9.750	−10.400	0.753
EFNO_x_	15	0.486	0.066	−3.956	7.754	0.236
EFSO_2_	15	−0.309	0.263	7.322	−6.581	0.095
EFTRS	14	−0.818	0.000	1.295	−1.378	0.669
EFPM_2.5_	13	−0.353	0.237	227.397	−229.783	0.125
Ponderosa Pine Needles						
EFCH_4_	14	−0.749	0.002	36.667	−36.982	0.560
EFC_2_H_2_	8	−0.940	0.001	13.744	−14.339	0.883
EFHCN	8	−0.973	0.000	5.772	−5.942	0.947
EFH_2_CO	8	−0.964	0.000	56.203	−58.635	0.930
EFCH_2_O_2_	8	−0.937	0.001	12.764	−13.483	0.878
EFNO_x_	14	−0.007	0.982	3.162	−0.182	0.000
EFSO_2_	14	−0.324	0.258	11.050	−10.388	0.105
EFTRS	13	−0.816	0.001	0.796	−0.823	0.666
EFPM_2.5_	13	−0.742	0.004	853.401	−893.968	0.551
Alberta						
EFCH_4_	11	−0.844	0.001	71.135	−72.938	0.712
EFC_2_H_2_	6	−0.129	0.807	0.863	−0.791	0.017
EFHCN	6	−0.882	0.020	4.997	−5.118	0.777
EFH_2_CO	6	−0.388	0.447	16.915	−17.017	0.151
EFCH_2_O_2_	6	−0.855	0.030	1.618	−1.672	0.730
EFNO_x_	11	−0.633	0.037	35.894	−33.954	0.400
EFSO_2_	11	−0.755	0.007	21.535	−21.162	0.570
EFTRS	7	−0.711	0.074	0.325	−0.325	0.505
EFPM_2.5_	11	−0.172	0.614	160.056	−158.687	0.029

1 MCE (modified combustion efficiency) = ΔCO_2_/(ΔCO+ΔCO_2_).

## Appendix B. Smoke Calibration of TSI Model 3321 APS

The accurate determination of PM_2.5_ from an APS using first principles requires each aerosol to be sampled representatively, sized accurately, counted with 100% efficiency, and have a known density (Peters, 2006). Past research has highlighted problems with these assumptions (Volckens and Peters, 2005; Peters 2006). The TSI Model 2231 (serial number 1270) used during this study was factory cleaned and calibrated just prior to deployment. During data processing from the TSI APS the instrument density factor was set to 1 and the Stokes correction was turned off. A series of 40 1 h static burns were conducted from April 12–24, 2021, at the USDA Forest Service Fire Sciences Laboratory by the study team using Ponderosa pine needles and fine woody debris fuels to test the performance of numerous commercially available instruments and sensors in smoke (manuscript in preparation). As part of that work just prior to the stack burns described in this paper, various smoke calibration approaches were investigated for the TSI Model 3321 APS and Teledyne API (San Diego, CA, USA) Model T640 scattered light spectroscopy U.S. EPA Federal Equivalent Method (*FEM*) instruments versus the mean of three collocated Tisch Environmental (Cleves, OH, USA) model TE-WILBUR filter-based PM_2.5_ U.S. EPA Federal Reference Method (FRM) samplers.

Evaluation of the temporal response characteristics versus CO reference measurements demonstrated the TSI APS provided faster aerosol response that better matched the structure of the time series data than the API T640 during both static and stack burns. Therefore, the TSI APS was selected for use in calculating PM_2.5_ emission rates. Numerous polynomial calibration models (e.g., cubic, inverse, linear, power, quadratic) were evaluated for fit and accuracy from various size APS size bins. A power model of FRM versus APS total particulate matter provided the best fit (Appendix Figure B.1) and accuracy (83.2 ± 14.3) (mean ± standard deviation). The fact that the total PM mass model (Appendix Figure B.1) was better fit than the APS PM_2.5_ model (Appendix Figure B.2) was perhaps due to lower APS counting efficiencies for smaller particles over various burn conditions that also resulted in lower overall accuracies (68.9 ± 66.6).
